# Prevalence of pretreatment HIV drug resistance in key populations: a systematic review and meta‐analysis

**DOI:** 10.1002/jia2.25656

**Published:** 2020-12-24

**Authors:** Virginia Macdonald, Lawrence Mbuagbaw, Michael R Jordan, Bradley Mathers, Sharon Jay, Rachel Baggaley, Annette Verster, Silvia Bertagnolio

**Affiliations:** ^1^ Department of HIV, Hepatitis, and Sexually Transmitted Diseases World Health Organization Geneva Switzerland; ^2^ Department of Health Research Methods, Evidence and Impact McMaster University Hamilton ON Canada; ^3^ Biostatistics Unit Father Sean O’Sullivan Research Centre St Joseph’s Healthcare‐Hamilton Hamilton ON Canada; ^4^ Division of Geographic Medicine Tufts Medical Center Boston MA USA; ^5^ Department of Public Health and Community Medicine Tufts University School of Medicine Boston MA USA; ^6^ Tufts Center for Integrated Management of Antimicrobial Resistance (CIMAR) Boston MA USA; ^7^ Levy Center for Integrated Management of Antimicrobial Resistance (CIMAR) Boston MA USA

**Keywords:** men who have sex with men, sex workers, people who inject drugs, prisoners, transgender, HIV drug resistance

## Abstract

**Introduction:**

WHO’s 2019 report on HIV drug resistance (HIVDR) documents a high prevalence of pretreatment drug resistance (PDR) among populations initiating first‐line antiretroviral therapy (ART) in low‐ and middle‐income countries (LMIC). However, systematic evidence on the prevalence of PDR among key populations remains limited. We performed a systematic review to characterize levels of PDR in key population groups and compared them to levels of PDR in the “general population” across different geographical regions.

**Methods:**

Ten electronic databases were searched for papers published until February 2019 that included predefined search terms. We included studies that reported the number of successfully tested genotypes and the number of genotypes with drug resistance mutations among antiretroviral therapy treatment naïve people, recently infected people, or people initiating first‐line ART from key populations. To assess the prevalence of PDR for each key population, we pooled estimates using random‐effects meta‐analysis of proportions. Where possible, we computed the differences in the odds of PDR (any, and by drug class) present in each key population compared to the “general population”. The I^2^ statistic (a measure of heterogeneity between studies) is reported.

**Results and discussion:**

A total of 332 datasets (from 218 studies) and 63,111 people with successful HIVDR genotyping were included in the analysis. The pooled prevalence estimate of any PDR was high among men who have sex with men (13.0%, 95% CI 11.0 to 14.0%, I^2^ = 93.19), sex workers (17.0%, 95% CI 6.0 – 32.0, I^2^ = 87.31%) and people in prisons (18.0%, 95% CI 11.0 to 25.0, I^2^ = 70.18%), but less so among people who inject drugs (7.0%, 95% CI 5.0 to 10.0, I^2^ = 90.23). Overall, men who have sex with men were more likely to carry any PDR compared to the “general population,” a finding which was statistically significant (odds ratio (OR) 1.28, 95% CI 1.13 – 1.46, I^2^ 48.9%).

**Conclusions:**

High prevalence of PDR found in key populations highlights the need to increase access to effective first‐line HIV treatment. The low prevalence of nucleotide reverse transcriptase inhibitor (NRTI) PDR suggests that current WHO recommendations for pre‐exposure prophylaxis (PrEP) regimens will remain effective and can be scaled up to prevent new HIV infections in high‐risk groups.

## 1 INTRODUCTION

The Joint United Nations Programme on HIV/AIDS (UNAIDS) estimates that 26 million people worldwide were receiving antiretroviral therapy (ART) for HIV treatment by the end of 2019, an increase of approximately 9 million since the end of 2015. The impressive global scale‐up of ART led to a near 39% reduction in AIDS‐related mortality between 2010 and 2019. Nonetheless, as of 2019, only 59% of all people living with HIV had suppressed viral loads [1]. Some degree of HIV drug resistance (HIVDR) can be expected to emerge and be transmitted in populations receiving ART even when adherence to therapy and retention are maximally supported. The term *pretreatment HIV drug resistance* (PDR) applies to HIVDR detected in individuals initiating ART regardless of prior antiretroviral (ARV) drug exposure(s) (e.g. prior ART followed by default from care, pre‐exposure prophylaxis (PrEP), post‐exposure prophylaxis (PEP)); thus, PDR may either be transmitted at time of initial infection or acquired by prior exposure to ARV drugs.

The World Health Organization’s (WHO’s) 2019 HIVDR report documents a high prevalence (≥10%) of PDR to efavirenz (EFV) and/or nevirapine (NVP) among adults initiating first‐line ART in 12 of 18 low‐ and middle‐income countries (LMIC) that were reporting results from nationally representative surveys [2]. These surveys, however, are not designed to yield PDR estimates stratified by HIV risk group and thus do not provide information on PDR in key populations, defined by the United Nations as men who have sex with men, people who inject drugs, sex workers, transgender people and people in prisons.

In lower‐, middle‐ and high‐income countries, key populations are disproportionately affected by HIV. Key populations are often isolated and discriminated against, and in many countries, their behaviours are criminalized. Despite global efforts to eliminate AIDS as a public health threat by 2030, the proportion of new infections that occur in key populations has steadily increased over the past decade [3]. UNAIDS estimated that in 2019 62% of all new HIV infections worldwide occurred in key populations and their sexual partners [4].

Factors such as limited access to HIV prevention methods as well as HIV testing and treatment services increase the risk of HIV acquisition as well as HIV transmission in members of key populations. For example less than 1% of people who inject drugs live in settings with high coverage of opioid substitution therapy (OST) and needle/syringe programmes (>200 needle‐syringes distributed per person who injects drugs and > 40 OST recipients per 100 persons who inject drugs )[5]. In South Sudan, Zimbabwe and Madagascar, less than one‐half of sex workers report receiving combined HIV prevention services in the past three months [6]. Members of key populations in LMIC may be unaware of their HIV status and a large proportion who do know that they have HIV infection may not be receiving treatment [7, 8, 9, 10, 11, 12].

There is limited systematic evidence about the prevalence of PDR in key populations. A previous systematic review, which included data published up to 2013, was restricted to men who have sex with men and people who inject drugs [13]. This publication documented a prevalence of any PDR (defined as resistance to one or more nucleotide reverse transcriptase inhibitors (NRTIs), and/or non‐nucleotide reverse transcriptase inhibitors (NNRTIs) and/or protease inhibitors (PIs)) above 10% in men who have sex with men and people who inject drugs, particularly in North America and Europe. Men who have sex with men were found to have a higher prevalence of any PDR compared to people who inject drugs and the “general population.” Notably, while an increasing prevalence of PDR over time was observed among men who have sex with men in lower‐income countries, levels were relatively stable in this population in upper‐income countries.

The presence of PDR in key populations has important implications for the choice of first‐line HIV treatment and may provide an improved understanding of potential reasons for treatment failure in these populations. Furthermore, with increasing numbers of men who have sex with men accessing PrEP for HIV prevention, the prevalence and patterns of HIVDR observed among members of this key population may have implications for the choice of optimal antiretroviral regimens for both the prevention and treatment of HIV in men who have sex with men.

We performed a systematic review and meta‐analysis to characterize levels of PDR in different key populations and compared them with levels of PDR in the “general population” in different geographical regions.

## 2 METHODS

### 2.1 Search strategy

In February 2019, the following 10 electronic databases were searched for papers published between January 1997 and February 2019 that included the pre‐defined search terms described below: PubMed, Scopus, WHO Global Health Libraries, Ovid Global Health, Sociological Abstracts, PsycINFO, EMBASE and POPLINE. Secondary reference searching was conducted on all studies included in the review. Furthermore, selected experts in the field were contacted to identify additional relevant articles not identified through other search methods. Search terms related to HIV, drug resistance, HIV treatments and key populations were used to develop a search strategy. See appendix.

### 2.2 Screening

We included studies that reported the number of genotypes successfully tested and the number of genotypes with drug resistance mutations among antiretroviral therapy treatment naïve people, recently infected people or people initiating first‐line ART from one of the following key populations: people who inject drugs, men who have sex with men, transgender people, sex workers or people in prisons. Eligible studies were expected to quantify the number of genotypes in at least one key population.

Eligible studies reported resistance using Sanger sequencing. Studies using ultra‐sensitive HIVDR detection methods (i.e. next‐generation sequencing) were included with data reported at threshold of 20% to approximate the lower limit of detection of Sanger sequencing. Studies reporting resistance using point mutations assays were excluded.

**Figure 1:**
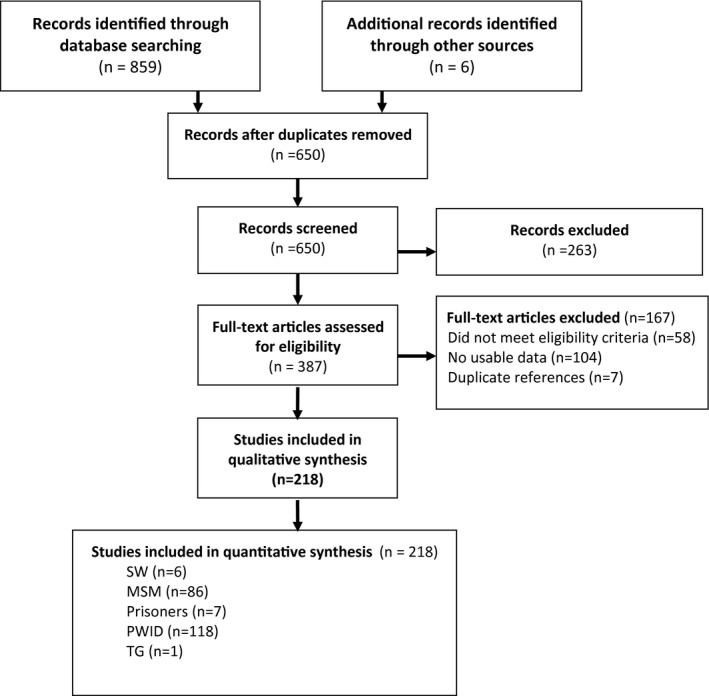
Flow chart showing study selection.

We first screened the titles and abstracts of studies for relevance and then pulled the full‐text articles of the potentially relevant ones for data abstraction. The screening and study selection process are outlined in PRISMA diagram (see Figure 1).

### 2.3 Data abstraction

We extracted the following variables: year of publication, years of data collection, the country in which the study was performed, prior exposure to antiretroviral therapy, total sample size, number belonging to each key population, number of people enrolled, number of people with successfully tested genotypes, and number of genotypes with resistance mutations (any PDR, and PDR by drug class). To allow intra‐study comparison between the prevalence of PDR in the “general population” versus key populations, we also extracted drug resistance prevalence information in the “general population” when it was reported in the same study.

The midpoint of data collection was computed as the median year during which data were collected. Individuals belonging to more than one key population were included in each relevant population group. Studies in which exposure to antiretroviral drugs was unclear or unspecified were excluded. The mutation list used by the author(s) was also recorded (WHO SDRM list [14]; Stanford HIVdb [15]; IAS‐USA [16]; ANRS [17]; other/unknown). Data were extracted from the abstracts if the full texts were not available. We categorized the studies according to their key population(s), WHO region (Africa, Europe, South East Asia, Western Pacific, Americas and Eastern Mediterranean) [18], World Bank income level [19], and whether they included HIVDR data on the “general population.” This gave rise to two sets of studies, the first yielding prevalence information in key populations only, the second with data supporting within‐study comparisons of the prevalence of PDR between key populations and the “general population,” the second set being a subset of the first one.

Screening and data abstraction were done in duplicate by LM, TA, FM, DL or AL. Discrepancies were resolved by consensus or by arbitration from VM or SB.

### 2.4 Risk of bias

The risk of bias was assessed using an adapted version of a tool proposed by Hoy et al. for the risk of bias in prevalence studies. This tool allows an assessment of risk of bias based on the representativeness of the sample, the sampling frame, sampling technique, response bias, the use of proxies, case definition, validity of measurements, uniformity of data collection, the prevalence period and the appropriateness of the numerator and denominator. An overall judgement was made of high, low or moderate risk of bias based on an appraisal of these items [20].

### 2.5 Data analysis

To assess the prevalence of PDR for each key population, we pooled estimates using random‐effects meta‐analysis of proportions. The variances were stabilized using the Freeman‐Tukey double arcsine transformation. Weighted pooled estimates were computed and then back‐transformed. Exact confidence intervals were computed by inverting the equal‐tailed test based on the binomial distribution. Pooled estimates were computed using the Dersimonian and Laird method based on the transformed values and their variances. In studies where there were zero individuals with drug resistance reported in a population, the proportion was estimated as 1/4n, where n was the total number of successful genotypes [21]. For example in a study with 50 individuals contributing resistance data, but zero genotypes with drug resistance, the prevalence will be computed as 1/(4*50)=0.005. This is a very small fraction that would not inflate our prevalence estimate but would allow us to incorporate the denominator of 50 in our analysis. The proportions (%), 95% confidence intervals, p‐values and I^2^ values are reported. The I^2^ ranges from 0 to 100, indicates the percentage of variance in the estimate due to heterogeneity.

WHO’s threshold for which NNRTI drugs (due to low barrier of resistance) are not recommended as a component of first‐line ART is NNRTI PDR prevalence of ≥ 10% [22]. For purposes of comparison, we used this threshold to define PDR prevalence in any group and for any drug class as “high” [22]. Drug resistance was reported according to the definition used by study authors.

For the “within‐study” comparisons, we computed the odds of having individuals carrying drug resistance (any drug resistance and by drug class) in each key population compared to the “general population”. Odds ratios were pooled and grouped by country income level and WHO region. We conducted a random‐effects meta‐analysis using the Mantel–Haenszel approach. Heterogeneity was assessed using the I^2^ statistic. The results are reported as odds ratios, 95% confidence intervals, p‐values and I^2^ [23].

To facilitate data analysis, given that some studies reported on more than one key population and some participants belonged to more than one key population group, we broke the data into homogenous data sets for each key population. For example studies that reported the prevalence of drug resistance in sex workers and people who inject drugs would be analysed as two datasets; a participant who was a sex worker who injected drugs would thus be counted in both datasets.

Statistical analysis was performed in Stata version 15.1 (StataCorp, USA), using the *metaprop* and *metareg* commands [24, 25].

## 3 RESULTS AND DISCUSSION

### 3.1 Study characteristic

A total of 332 datasets (from 218 studies) and 63,111 participants with successful HIVDR genotyping were included in the analysis; the majority of data are from men who have sex with men (53.9% of datasets and 83.6% of participants), followed by people who inject drugs (37.7% and 14.8% respectively), sex workers (5.4% and 0.8% respectively), people in prisons (2.7% and 0.7% respectively) and transgender people (0.3% and 0.1% respectively). As previously mentioned, some participants contributed data to more than one key population and some studies contributed data to more than one group (Table 1).

**Table 1:** Number of datasets and number of participants with successful genotyping included in the analysis

Studies reporting only key populations^a^			
Key population	Number of datasets (%)	Number of participants with successful genotyping (%)	Median sampling year (range)
Sex workers	18 (5.4)	518 (0.8)	2009 (2001 to 2017)
Men who have sex with men	179 (53.9)	52731 (83.6)	2007 (1988 to 2018)
Prisoners	9 (2.7)	470 (0.7)	2006 (2000 to 2013)
People who inject drugs	125 (37.7)	9330 (14.8)	2006 (1990 to 2017)
Transgender people	1 (0.3)	62 (0.1)	2008 (2008 to 2008)
**Total**	332 (100.0)	63111 (100.0)	2007 (1988 to 2018)

Studies reporting key populations and the general population^a^				
Key population	Number of data sets (%)	Number of participants with successful genotyping (%)		Median sampling year (range)
		Key population	General population	
Sex workers	5 (4.5)	92 (0.3)	336 (1.0)	2012 (2006 to 2012)
Men who have sex with men	60 (54.5)	24880 (88.2)	20068 (61.7)	2012 (2001 to 2018)
People who inject drugs	45 (41.0)	3247(11.5)	12137 (37.3)	2013 (2001 to 2018)
**Total**	110 (100.0)	28219 (100.0)	32541 (100.0)	2012 (2001 to 2018)

^a^ Some participants contributed to more than one key population and some studies contributed data to more than one category.

Thirty‐seven eligible studies (110 data sets) reported PDR data in one or more key populations as well as in the “general population” and were included in the analysis of “within‐study comparison”: 60, 45 and 5 datasets reported PDR data in both the “general population” and men who have sex with men, people who inject drugs and sex workers respectively (Table 1).

Half of the data on key populations (117 studies, 53.7% of all studies included) came from high‐income countries, followed by upper middle‐income countries (78 studies, 35.8%). No study on sex workers came from a high‐income country. The WHO European region contributed the most studies (75, 34.4%) followed by the Americas (69, 31.7%), Western Pacific (48, 22.0%), South East Asia (13/6.0%), Africa (6/2.8%) and Eastern Mediterranean regions (3/1.4%). Four studies (1.8%) included countries belonging to more than one WHO region. See Table S1 Distribution of datasets by key population and income level, Table S2 Distribution of datasets by key population and region and Table S3 Distribution of participants by key population and region.

### 3.2 Risk of bias

**Figure 2:**
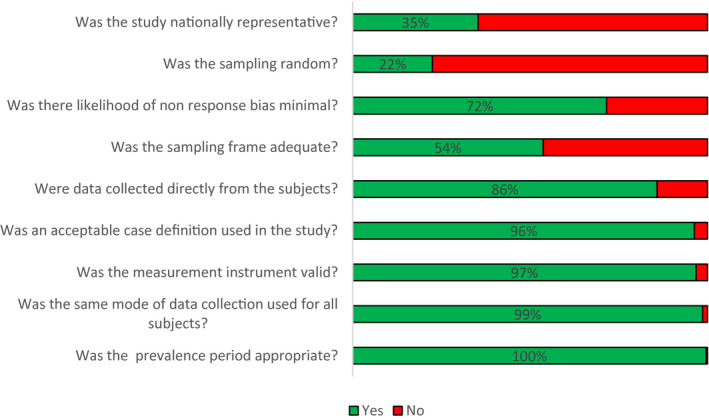
Risk of bias in included studies (n = 218).

Overall, risk of bias was high in 69 studies (31.7%), moderate in 34 (15.6%) and low in 115 (52.8%). The two most common concerns were that the included populations were not nationally representative (64.4%), and that inclusion of participants was not random (77.8%). Only in 120 studies (53.3%) was the sampling frame a true or close representation of the population of interest. A full description of risk of bias judgement is reported in Table S4 and is summarized in Figure 2.

### 3.3 Pooled prevalence of pretreatment drug resistance in different key population groups

Overall, the pooled prevalence estimate of any PDR was high (> 10%) among men who have sex with men (13.0%, 95% CI 11.0% to 14.0%, I^2^ = 93.19), sex workers (17.0%, 95% CI 6.0 – 32.0, I^2^ = 87.31%) and people in prisons (18.0%, 95% CI 11.0 to 25.0, I^2^ = 70.18%), but not in people who inject drugs (7.0%, 95% CI 5.0 to 10.0, I^2^ = 90.23%), although there was considerable heterogeneity among studies in all groups.

**Figure 3:**
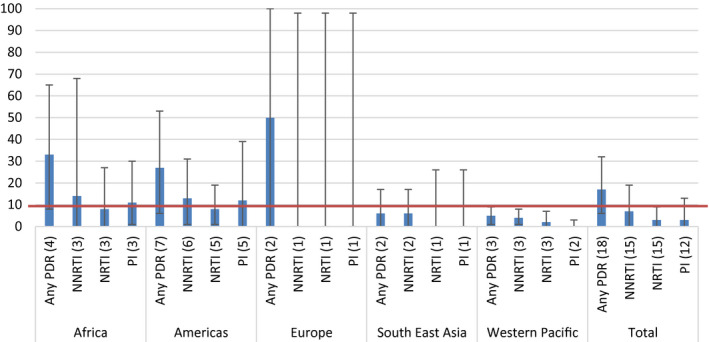
Estimated pooled prevalence of PDR among sex workers, by drug class and by WHO region (number of datasets). 10% NNRTI PDR WHO‐recommended threshold for switch to second line treatment is marked.

Regional analysis showed that for sex workers, a high prevalence of any PDR was found in Africa (33.0%, 95% CI 8.0 to 65.0, I^2^ = 95.64%), the Americas (27.0%, 95% CI, 6.0 to 53.0, I^2^ = 71.13%) and Europe (50.0%, 95% CI 0.0 to 100.0, I^2^ = not estimable (NE)); although based on few studies and with considerable heterogeneity between studies. In sex workers, pooled prevalence of resistance to NNRTI drugs was 7% (95% CI 0.0 to 19.0, I^2^ = 86.78%), to NRTI drugs 3.0% (95% CI 0.0 to 9.0, I^2^ = 61.86%) and to PI 3.0% (95% CI 0.0 to 13.0, I^2^ = 76.86%). In Africa and the Americas the overall high prevalence of any PDR was driven by the high prevalence of resistance to the NNRTI and PI drug class, although in Europe, due to the small number of eligible studies, it was not possible to calculate PDR prevalence by drug class (Figure 3).

**Figure 4:**
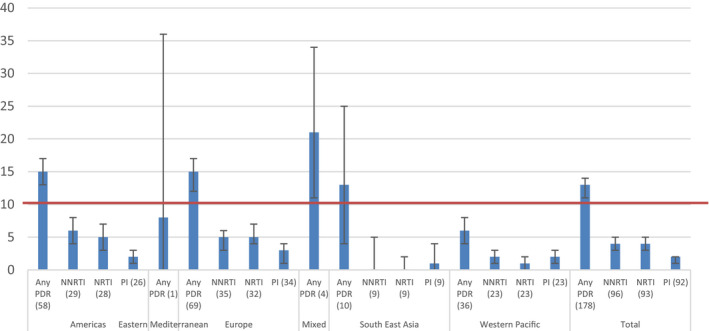
Estimated pooled prevalence of PDR among men who have sex with men, by drug class and by WHO region (number of datasets) 10% NNRTI PDR WHO recommended threshold for switch to second line treatment is marked.

For men who have sex with men, high prevalence of any PDR was found in the Americas (15.0%, 95% CI 13.0 to 17.0, I^2^ = 91.38%), Europe (15.0%, 95% CI 12.0 to 17.0, I^2^ = 92.88%) and South East Asia (13.0%, 95% CI 4.0 to 25.0, I^2^ = 84.71%). The subset of studies, which allowed calculation of PDR by drug class and by region for men who have sex with men, did not show high prevalence in any region for men who have sex with men (Figure 4). In men who have sex with men, the pooled prevalence of resistance to NNRTI drugs was 2% (95% CI 1.0 to 3.0), NRTI drugs 1.0% (95% CI 0.0 to 2.0) and PI 2.0% (95% CI 1.0 to 3.0).

**Figure 5:**
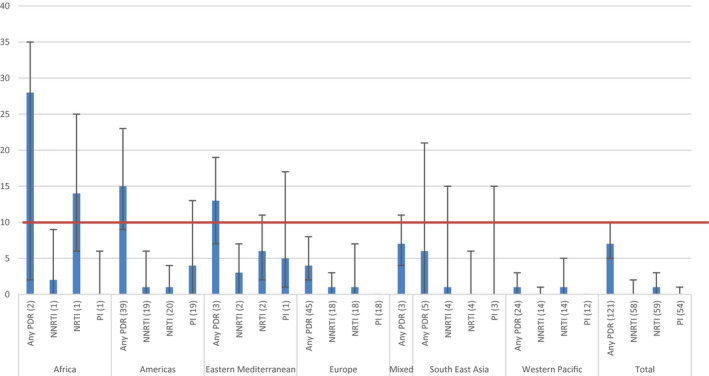
Estimated pooled prevalence of PDR among people who inject drugs, by drug class and by WHO region (number of datasets) 10% NNRTI PDR WHO‐recommended threshold for switch to second line treatment is marked.

While the overall prevalence of any PDR among people who inject drugs was < 10%, levels were high in Africa (28.0%, 95% CI 2.0 to 35.0, I^2^ = NE), in the Americas (15.0%, 95% CI 9.0 to 23.0, I^2^ = 93.40%) and the Eastern Mediterranean (13.0%, 95% CI 7.0 to 19.0, I^2^ = NE). In people who inject drugs, pooled prevalence of resistance to NNRTI drugs was 0% (95% CI 0.0 to 2.0), to NRTI drugs 1.0% (95% CI 0.0 to 3.0) and to PI 0.0% (95% CI 0.0 to 1.0). In the African region, results from 1 study showed the high prevalence of any PDR driven by resistance to the NRTI drug class (14.0%, 95% CI 6.0 to 25.0, I^2^ = NE) (Figure 5).

**Figure 6:**
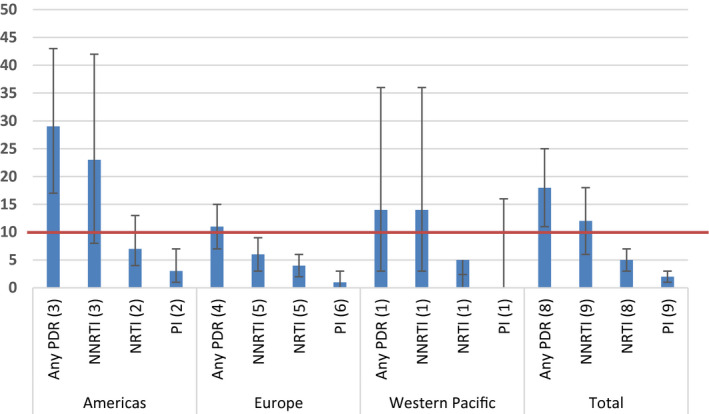
Estimated pooled prevalence of PDR among people in prisons, by drug class and by WHO region (number of datasets) 10% NNRTI PDR WHO‐recommended threshold for switch to second line treatment is marked.

Among people in prisons, the prevalence of any PDR was high in all regions with available data, and highest in the Americas (29.0%, 95% CI17.0 to 43.0, I2 = NE) followed by Europe (11.0%, 95% CI 7.0 to 15.0, I2 = 0.0%), and the Western Pacific (14.0%, 95% CI 3.0 to 26.0, I2 = NE) (Figure 6). In prisoners, the pooled prevalence of resistance to NNRTI drugs was 12% (95% CI 6.0 to 18.0), NRTI drugs 5.0% (95% CI 3.0 to 7.0) and PI 2.0% (95% CI 1.0 to 3.0).

See also, Table S5 Prevalence of PDR by region and Table S6 Prevalence of PDR by income level.

### 3.4 Within study comparison: key populations vs “general population”

**Figure 7:**
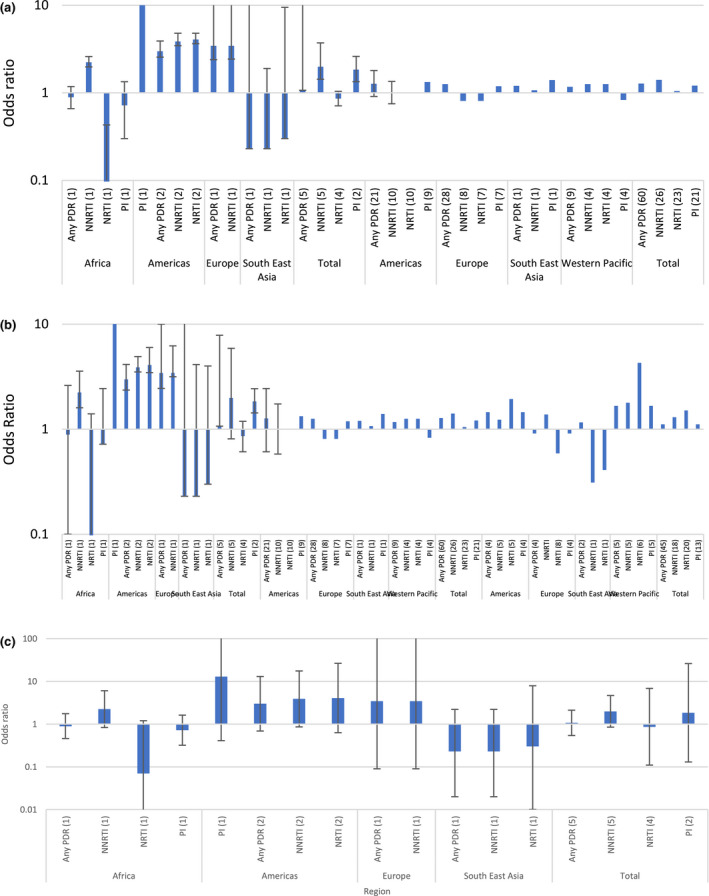
Odds ratios PDR key population versus “general population” (**A**) Sex workers (**B**) Men who have sex with men (**C**) People who inject drugs.

Overall, men who have sex with men were more likely to carry any PDR compared to the “general population,” a finding which was statistically significant (odds ratio (OR) 1.28, 95% CI 1.13 – 1.46, I^2^ 48.9%). However, results do not show which drug class drove this finding as there was no statistically significant increase in the odds of PDR among men who have sex with men compared to the “general population” for NRTI, NNRTI or PI drug classes. Results showed higher odds of PDR among men who have sex with men compared to the “general population” in high‐income countries (OR 1.29, 95% CI 1.13 to 1.49, I^2^ 39.0%), in Europe (OR 1.29, 95% CI 1.05 to 1.52, I^2^ 49.5%) and the Americas (OR 1.27, 95% CI 1.04 to 1.56, I^2^ 41.7%) (Figure 7).

There was no statistically significant increase in the odds of PDR compared to the “general population” among sex workers (OR 1.07, 95% CI 0.54 to 2.12, I^2^ 7%) or people who inject drugs (OR 1.11, 95% CI 0.86 to 1.44, I^2^ 52.4%). There were insufficient data to compare PDR prevalence estimates among people in prisons to estimates of PDR in the “general population” as no studies included both groups.

## 4 DISCUSSION

This systematic review includes 63111 participants with successful genotyping from 218 studies and 332 datasets including data collected between 1988 and 2019 (median 2007) and aims to investigate the prevalence of PDR in different key populations and compare it, where possible, to the prevalence observed in the “general population”.

Our findings suggest that the prevalence of PDR is high, exceeding 10% among men who have sex with men, sex workers and people in prisons. Additionally, men who have sex with men are more likely to carry drug resistance mutations compared to the “general population”, although the odds were only slightly higher.

The levels of PDR among key populations observed in our review appear to be higher than those reported by Gupta et al in the “general population”[21]. In their systematic review of PDR among treatment naive or individuals starting ART in the “general population” in LMIC Gupta and colleagues reported a prevalence of PDR (defined as resistance to ARVs of any drug class) in Southern Africa increasing from 1.3% before 2005 to 12.2% between 2014 and 2016 and in Western Africa, increasing from 4% before 2005 to 4.1% between 2014 and 2016. In our review, we found that among key populations in Africa the prevalence of any PDR was 33% in sex workers and 28% in people who inject drugs. In Asia, Gupta et al. found that the prevalence of any PDR in treatment naive people in the “general population” was 2.6% before 2005 increasing to 5.5% between 2014 and 2016 while we found a prevalence of 13% among men who have sex with men in South East Asia and 14% among people in prison in the Western Pacific region. Our study had a wider range of sampling years (1988 to 2018) compared to Gupta et al. (2001 to 2016); however, the median data collection date was similar (2008 for Gupta et al. and 2007 for our review). The earliest sampling years for men who have sex with men and people who inject drugs were earlier than for other groups which increases the number of available datasets for men who have sex with men and people who inject drugs but could also influence the results with greater exposure to older ARVs in these groups, particularly NRTIs.

We expected to see the high prevalence of PDR to the NNRTI drug class, as found in the “general population.” However, high levels of resistance to NNRTI drugs were only seen in people in prison and sex workers in Africa and the Americas and not in other key populations and other regions. Overall, our findings cannot explain which drug class was driving the high PDR prevalence found in key populations. This is likely due to study heterogeneity and other limitations. The confidence intervals for PDR prevalence in our review were sometimes wide and could encompass a higher estimate of PDR to NNRTIs as found in the other reviews, which reported overall population prevalence not disaggregated by key population.

When comparing the prevalence of PDR in key populations to the “general population” in a subset of 110 studies allowing within‐study comparison, our findings suggest that men who have sex with men in high‐income countries had greater likelihood of having a drug‐resistant virus compared to the “general population.” This observation is likely due to the relatively high coverage of HIV treatment among this population in Europe and the Americas; for example, an estimated 90.3% of all men who have sex with men living with HIV were receiving treatment for HIV in Canada in 2018; in Spain, 76.8% were receiving treatment in 2016 and in Switzerland, 87.7% were receiving treatment in 2018 [26]. In comparison, while data are limited and likely to be incomplete, treatment access and coverage for men who have sex with men are likely to be considerably lower in other regions of the world. For example, country reports to UNAIDS show that only 3.7% of men who have sex with men living with HIV in Ghana were receiving treatment in 2017, 14.1% in Togo in 2018 and 28.1% in South Africa in 2017 [27]. This low ART coverage may explain the lower than expected circulation of transmitted resistance within these groups.

Although our review looked at a large number of studies and included an assessment of the risk of bias, it has several limitations. First, many of the included studies were not designed to specifically estimate the prevalence of HIVDR in key populations; thus, data were drawn from subsets of participants who self‐identified as a member of a given population. As such, the risk of bias is high or medium in several studies and generalizability is limited. Our full risk of bias assessment highlights further concerns with individual studies. For example, many studies were dropped because the participant’s probable route of transmission or membership in a key population was not reported, thus potentially resulting in reporting and selection biases. Second, the prevalence of PDR by drug class was not reported uniformly across all studies. Third, despite breaking down the data by key population, income level as defined by the World Bank, and WHO region, substantial unexplained heterogeneity persists. This heterogeneity (as indicated by high I^2^ values) may be due to differences in study design, the choice of effect measure or the large number of studies [28]. While this heterogeneity is incorporated into the random effects models, it is still concerning. Examining the data only from studies that reported all three drug classes and studies with a large sample size did not materially change the results. We therefore advise caution in the interpretation of these findings and suggest that they be viewed as the “average” prevalence across studies. Considering this, we have reported the median, minimum and maximum values of prevalence (see supplementary material) Finally, data included are from reports published over slightly more than a 20‐year period, with most recent papers from February 2019 and while we do not expect it would substantially change the pooled results, additional reports of PDR among key populations may have been published since then.

While we included studies reporting PDR disaggregated by drug class and population, we did not have individual sequence data in file format for analysis so our results are based on data analysed and reported by the individual study authors. Studies lacked standardized methods to interpret resistance which may limit comparability of data and may result in an underestimation or overestimation of the levels of resistance reported in the review.

In some regions, there was a lack of data for certain key population groups: for example, there were no studies of sex workers from high‐income countries, no studies of men who have sex with men from low‐income countries and no studies of transgender people or prisoners in either low or lower middle‐income countries (please see supplementary material). One reason why many studies in Europe and the US provide data about men who have sex with men and people who inject drugs, but not sex workers is because the categorization depends on the reported route of transmission (heterosexual, homosexual, parenteral), which is included in case report forms in countries where case surveillance systems are used, for example the United States and Europe, but not so frequently in LMIC. In general, there were less data available from low‐income settings.

Pooling global data and presenting results by key population groups may mask regional differences in the lives and behaviours of people in different regions. In particular, as above, in some regions there were few or no data sets available for analysis and conclusions drawn about populations in these regions based on global data should be interpreted with some caution. There is a need for additional data about PDR in key populations from LMIC, with disaggregation by key population in surveillance systems where possible.

Given the high prevalence estimates of PDR among key populations, attention should be paid to strengthened monitoring of viral load and early detection of treatment failure in these groups, and rapid switching to recommended second‐line regimens when necessary. Appropriate, highly potent regimens should be provided and should be informed by local epidemiology and resistance patterns among new treatment initiators in key populations. In this review we did not estimate the prevalence of PDR to integrase strand transfer inhibitors (INSTIs); although not excluded from the search, we did not find reports of PDR to INSTI in key populations in any of the included studies. However, INSTIs are a key component of globally recommended regimens for HIV treatment and the PDR we identified against older drug classes may not be clinically relevant. The WHO recommended first‐generation agent, dolutegravir (DTG), combined with two NRTIs, is highly potent and is associated with less risk of discontinuing treatment compared to NNRTI‐based ART [29]. DTG also has a high genetic barrier to developing HIVDR than EFV or NVP [30] and to date the transmission of INSTI resistance mutations is extremely rare [31, 32, 33]. Key populations should have equal access to DTG as a first‐line regimen for the treatment of HIV.

PrEP is the use of antiretroviral medication to prevent the acquisition of HIV infection by uninfected persons. Currently, the availability of PrEP outside upper‐income countries is low, but PrEP scale‐up is increasing in many LMIC. For people at high risk of HIV infection, WHO recommends PrEP regimens which contain tenofovir (TDF) alone or tenofovir in combination with emtricitabine/lamivudine (XTC), – two NRTIs which are also recommended as components of first‐line ART. Modelling studies suggest that by preventing HIV acquisition, PrEP scale‐up would result in overall lower levels of drug resistance [34]. In addition, PrEP programmes report only a few cases of HIVDR in people who acquire HIV while taking PrEP (as most people who seroconvert do so while they are not taking PrEP) [34]. Despite this evidence, some policy makers remain concerned about the potential for a significant increase in TDF/XTC resistance resulting from the scaled‐up use of PrEP [35, 36, 37, 38]. The low levels of NRTI PDR found in our review are consistent with levels observed in nationally representative surveys of PDR where the prevalence of TDF or XTC resistance in ARV drug naïve people was generally very low [29]. Furthermore, consistent with our findings, a review of the safety and effectiveness of oral PrEP [39] did not find high levels of NRTI drug resistance in any population or region, nor were there significantly higher odds of key population groups having PDR to NRTI drugs than in the “general population.” These findings suggest that within networks of men who have sex with men there are low rates of transmitted drug resistance to TDF/XTC, although it should be acknowledged that individuals who default from HIV treatment may have drug resistance mutations archived in viral DNA, and while resistance fades rapidly from circulating RNA (PDR), it can emerge quickly with reintroduction of drug selective pressure.

This analysis supports the use of TDF containing PrEP to prevent HIV acquisition. Additional surveillance and studies of populations taking PrEP in LMIC are warranted as PrEP is scaled‐up in LMIC.

## 5 CONCLUSIONS

To the best of our knowledge, this is the first systematic review and meta‐analysis that has examined the global prevalence of PDR among all five United Nations defined key populations by drug class, region and country income level. The high prevalence of PDR found in some key populations highlights the need to increase access to effective first‐line HIV treatment in these groups. The low prevalence of PDR to NRTIs suggests that current WHO recommendations for PrEP regimens will remain effective and can be scaled up to prevent new HIV infections in high‐risk groups.

## COMPETING INTERESTS

No competing interests.

## AUTHORS’ CONTRIBUTIONS

VM was responsible for study protocol design, oversight and writing. LM was responsible for data analysis, figures and tables. BM and SJ were responsible for data abstraction and input to draft text. RB, AV and MJ provided input to protocol design and writing. SB was responsible for overall coordination.

## Supporting information


**Table S1.** Distribution of datasets by key population and income level (number of datasets and % by income level)
**Table S2.** Distribution of datasets by key population and region (number of datasets and % by WHO region)
**Table S3.** Distribution of participants by key population and region
**Table S4.** Assessment of risk of bias
**Table S5.** Prevalence of PDR by region
**Table S6.** Prevalence of PDR by income level
