# Supplementary material for: Prevalence of pretreatment HIV drug resistance in key populations: a systematic review and meta‐analysis
Source: J Int AIDS Soc. 2020 Dec 24;23(12):e25656. doi: 10.1002/jia2.25656 (PMC7758978; doi:10.1002/jia2.25656)
Supplement: Supplementary file 1 — Table S1. Distribution of datasets by key population and income level (number of datasets and % by income level) Table S2. Distribution of datasets by key population and region (number of datasets and % by WHO region) Table S3. Distribution of participants by key population and region Table S4. Assessment of risk of bias Table S5. Prevalence of PDR by region Table S6. Prevalence of PDR by income level [file JIA2-23-e25656-s001.docx]

**Supplemental information**

**Table 1: Distribution of datasets by key population and income level (number of datasets and % by income level)**

|  | Low | Low Middle | Upper Middle | High | Mixed | Total |
| --- | --- | --- | --- | --- | --- | --- |
| Sex workers n (%) | 2 (50.0%) | 7 (26.9%) | 8 (7.3%) | 1 (0.5%) | 0 (0.0%) | 18 (5.4%) |
| Men who have sex with men n (%) | 1 (25.0%) | 6 (23.1%) | 58 (53.2%) | 110 (58.8%) | 4 (66.7%) | 179 (53.9%) |
| Prisoners n (%) | 0 (0.0%) | 0 (0.0%) | 3 (2.8%) | 6 (3.3%) | 0 (0.0%) | 9 (2.7%) |
| People who inject drugs n (%) | 1 (25.0%) | 13 (50.0%) | 39 (35.8%) | 70 (37.4%) | 2 (33.3%) | 125 (37.7%) |
| Transgender people n (%) | 0 (0.0%) | 0 (0.0%) | 1 (0.9%) | 0 (0.0%) | 0 (0.0%) | 1 (0.3% |
| **Total** | **4** | **26** | **109** | **183** | **6** | **332** |

**Table 2: Distribution of datasets by key population and region (number of datasets and % by WHO region)**

|  | Africa | America | Eastern Mediterranean | Europe | South East Asia | Western Pacific | Mixed | Total |
| --- | --- | --- | --- | --- | --- | --- | --- | --- |
| Sex workers n (%) | 4 (66.7%) | 7 (6.4%) | 0 (0.0%) | 2 (1.6%) | 2 (11.8%) | 3 (4.6%) | 0 (0.0%) | 18 (5.4%) |
| Men who have sex with men n (%) | 0 (0.0%) | 57 (52.3%) | 1 (20.0%) | 71 (57.7%) | 10 (58.8%) | 36 (55.4%) | 4 (57.1%) | 179 (53.9%) |
| Prisoners n (%) | 0 (0.0%) | 4 (3.7%) | 0 (0.0%) | 4 (3.3%) | 0 (0.0%) | 1 (1.5%) | 0 (0.0%) | 9 (2.7%) |
| People who inject drugs n (%) | 2 (33.3%) | 40 (36.7%) | 4 (80.0%) | 46 (37.4%) | 5 (29.4%) | 25 (38.5%) | 3 (42.9%) | 125 (37.7%) |
| Transgender people n (%) | 0 (0.0%) | 1 (0.9%) | 0 (0.0%) | 0 (0.0%) | 0 (0.0%) | 0 (0.0%) | 0 (0.0%) | 1 (0.3%) |
| **Total** | **6** | **109** | **5** | **123** | **17** | **65** | **7** | **332** |

**Table 3: Distribution of participants by key population and region**

| **Key pop** | **Africa** | **America** | **Eastern Mediterranean** | **Europe** | **South East Asian** | **Western Pacific** | **Mixed** |
| --- | --- | --- | --- | --- | --- | --- | --- |
| **SW: n (%)*** | 236 (63.3) | 103 (0.4) | 0 (0.0) | 2 (0.0) | 34 (3.5) | 143 (1.3) | 0 (0.0) |
| **MSM: n (%)*** | 0 (0.0) | 24629 (86.4) | 13 (7.1) | 18215 (85.6) | 643 (66.6) | 8263 (77.7) | 968 (83.2) |
| **PWID: n (%)*** | 137 (36.7) | 3510 (12.3) | 170 (92.9) | 2818 (13.2) | 289 (29.9) | 2210 (20.8) | 196 (16.8) |
| **Prisoners: n (%)*** | 0 (0.0) | 206 (0.7) | 0 (0.0) | 243 (1.1) | 0 (0.0) | 21 (0.2) | 0 (0.0) |
| **TG: n (%)*** | 0 (0.0) | 62 (0.2) | 0 (0.0) | 0 (0.0) | 0 (0.0) | 0 (0.0) | 0 (0.0) |
| **Total: n (%)*** | 373 (100.0) | 103 (100.0) | 183 (100.0) | 21278 (100.0) | 966 (100.0) | 10637 (100.0) | 1164 (100.0) |

*Column percentages; SW: sex workers; MSM: men who have sex with men; PWID: people who inject drugs; TG transgender

**Search terms**

The following terms were adapted for entry into all computer databases.

**1: HIV/AIDS**

“HIV Infections” [MeSH] OR “HIV”[MeSH] OR “hiv”[tw] OR “hiv-1”[tw] OR “hiv-2”[tw] OR “hiv1”[tw] OR “hiv2”[tw] OR hiv infect*[tw] OR “human immunodeficiency virus”[tw] OR “human immunedeficiency virus”[tw] OR “human immuno-deficiency virus”[tw] OR “human immune-deficiency virus”[tw] OR ((human immun*) AND (“deficiency virus”[tw])) OR “acquired immunodeficiency syndrome”[tw] OR “acquired immunedeficiency syndrome”[tw] OR “acquired immuno-deficiency syndrome”[tw] OR “acquired immune-deficiency syndrome”[tw] OR ((acquired immun*) AND (“deficiency syndrome”[tw])) OR "Sexually Transmitted Diseases, Viral"[MeSH:NoExp]

**2: Drug resistance/Treatment failure**

"Drug Resistance"[Mesh] OR resistance[tw] OR “drug resistance”[tw] OR “drug resistant”[tw] OR resistant[tw] OR mutant[tw] OR mutation[tw] OR genotype[tw] OR genotyp*[tw] OR genotypic[tw] OR “virological failure”[tw] OR “treatment failure”[tw] OR "Treatment Failure"[Mesh] OR "Viral Load"[Mesh] OR “viral load” OR “transmitted resistance”[tw] or “pre-treatment drug resistance”[tw] OR naïve[tw] OR “transmitted drug resistance”[tw] OR “TDR” [tw]

**3:HIV treatment**

"Anti-HIV Agents"[Mesh] OR “ARV” or “ART” or “HIV treatment” OR “Antiretroviral” OR atazanavir OR darunavir OR dolutegravir OR fosamprenavir OR indinavir OR lopinavir OR saquinavir OR efavirenz OR enfuvirtide OR etravirine OR lamivudine OR maraviroc OR nevirapine OR raltegravir OR rilpivirine OR saquinavir OR tenofovir OR tipranavir OR “non-nucleoside reverse-transcriptase inhibitors” OR “NNRTI” OR “nucleoside reverse-transcriptase inhibitors” OR “NRTI” OR “HAART” OR “protease inhibitor” OR “boosted protease inhibitor” OR “PI” OR “bPI” OR “highly active antiretroviral therapy” OR “antiretroviral therapy” OR stavudine OR zidovudine OR nevirapine OR efavirenz OR antiretrovir*

**4: Key populations**

1. **Men who have sex with men and transgender**

“men who have sex with men”[ALL] OR "Homosexuality"[Mesh] OR bisexuality[Mesh] OR queer[ALL] OR “men-who-have-sex-with-men”[ALL] OR “MSM”[ALL] OR “gay”[ALL] OR “homosexual”[ALL] OR “homosexuals”[ALL] OR “bi-sexual”[ALL] OR LGB*[ALL] OR "Transgender Persons"[Mesh] OR transgender[ALL] OR transexualism[ALL] OR LBG*[ALL] OR “sexual minority”[TW] OR transsexual[ALL] OR “sexual orientation”[ALL]

1. **Sex workers**

"Prostitution"[Mesh] OR “sex worker”[ALL] OR “sex workers”[ALL] OR “commercial sex worker”[ALL] OR “ prostitute”[ALL] OR brothel*[ALL] OR escort[ALL] OR “commercial sex”[ALL] OR “ commercial sex worker”[ALL] OR “sex work”[ALL] OR prostitut*[ALL] OR prostitution[ALL] OR brothel*[ALL] OR escort[ALL]

1. **People who inject drugs**

“drug user”[ALL] OR “drug addict”[ALL] OR addict[ALL] OR “injecting drug user”[ALL] OR “injection drug user”[ALL] OR “injecting drug users”[ALL] OR “injection drug users”[ALL] OR “people who inject drugs”[ALL] OR “people who use drugs”[ALL] OR “substance abuse”[ALL] OR “substance misuse”[ALL] OR “substance use”[ALL] OR “substance use disorder”[ALL] OR “substance user[ALL] OR “PWID”[ALL] OR "Substance Abuse, Intravenous"[Mesh] OR addiction[ALL]

1. **Prisoners and other people in closed settings**

"Prisoners"[Mesh] AND "Prisons"[Mesh] OR “forced treatment”[ALL] OR “compulsory treatment”[ALL] OR detention[ALL] OR “mandatory detention”[ALL] OR “detention centre”[ALL] OR “detention center”[ALL] OR prison[ALL] OR prisoner[ALL] OR offender[ALL] OR jail[ALL] OR correctional[ALL] OR inmate*[ALL]

**Table 4: Assessment of risk of bias**

| **study** | **year** | **Nationally representative?** | **Good sampling frame?** | **Random selection?** | **Response bias** | **No proxy** | **Case definition** | **Valid reliable tool?** | **Same mode of data collection?** | **Prevalence period?** | **Numerator and denominator?** | **Overall judgement** |
| --- | --- | --- | --- | --- | --- | --- | --- | --- | --- | --- | --- | --- |
| Alexander | 1999 | Yes | Yes | No | Yes | Yes | Yes | Yes | Yes | Yes | Yes | Low |
| Alexiev | 2016 | Yes | Yes | No | No | No | Yes | Yes | Yes | Yes | Yes | Low |
| Ananworanich | 2015 | Yes | Yes | No | Yes | No | Yes | Yes | Yes | Yes | Yes | Low |
| Ananworanich | 2008 | No | No | No | No | Yes | Yes | Yes | Yes | Yes | Yes | High |
| Andersson | 2018 | Yes | Yes | Yes | Yes | No | Yes | Yes | Yes | Yes | Yes | Low |
| Andreani | 2011 | No | No | No | No | Yes | Yes | Yes | Yes | Yes | Yes | High |
| Archibald | 2005 | Yes | Yes | Yes | Yes | No | Yes | Yes | Yes | Yes | No | Low |
| Ariffin | 2014 | No | No | No | Yes | Yes | Yes | Yes | Yes | Yes | Yes | High |
| Audelin | 2011 | Yes | Yes | No | Yes | No | Yes | Yes | Yes | Yes | No | Low |
| Ávila-Ríos | 2015 | Yes | Yes | Yes | Yes | Yes | Yes | Yes | Yes | Yes | No | Low |
| Avila-Ríos | 2011 | No | No | No | Yes | Yes | Yes | Yes | Yes | Yes | Yes | Moderate |
| Babič | 2006 | No | No | No | No | Yes | Yes | Yes | Yes | Yes | Yes | High |
| Bannister | 2008 | Yes | Yes | No | Yes | Yes | Yes | Yes | Yes | Yes | No | Low |
| Barbosa de Medeiros | 2006 | No | No | No | No | Yes | Yes | Yes | Yes | Yes | Yes | High |
| Bermudez-Aza | 2011 | No | Yes | No | Yes | Yes | Yes | Yes | Yes | Yes | Yes | Low |
| Bezemer | 2004 | No | No | No | Yes | No | Yes | Yes | Yes | Yes | No | High |
| Bhusal | 2016 | No | No | No | Yes | Yes | Yes | Yes | Yes | Yes | No | Moderate |
| Bontell | 2012 | No | No | No | Yes | Yes | Yes | Yes | Yes | Yes | No | High |
| Bonuro | 2010 | Yes | Yes | Yes | Yes | No | Yes | Yes | Yes | Yes | Yes | Low |
| Booth | 2006 | Yes | Yes | Yes | Yes | Yes | Yes | Yes | Yes | Yes | Yes | Low |
| Brindeiro | 2003 | Yes | Yes | Yes | Yes | Yes | Yes | Yes | Yes | Yes | Yes | Low |
| Briones | 2001 | No | No | No | Yes | Yes | Yes | Yes | Yes | Yes | Yes | High |
| Brooks | 2013 | Yes | Yes | Yes | Yes | Yes | Yes | Yes | Yes | Yes | No | Low |
| Buchacz | 2015 | Yes | No | No | Yes | No | Yes | No | Yes | Yes | Yes | Moderate |
| Budambula | 2015 | Yes | Yes | No | Yes | Yes | Yes | Yes | Yes | Yes | No | Low |
| Burchell | 2011 | Yes | Yes | Yes | Yes | Yes | Yes | Yes | Yes | Yes | Yes | Low |
| Cardoso | 2009 | No | Yes | Yes | Yes | Yes | Yes | Yes | Yes | Yes | Yes | Low |
| Carobene | 2014 | Yes | Yes | Yes | Yes | Yes | Yes | Yes | Yes | Yes | Yes | Low |
| Carvalho | 2011 | Yes | Yes | Yes | Yes | Yes | Yes | Yes | Yes | Yes | Yes | Low |
| Cavalcanti | 2012 | No | No | No | No | Yes | Yes | Yes | Yes | Yes | Yes | High |
| Cecchini | 2015 | No | No | No | No | No | No | Yes | No | Yes | Yes | High |
| Chaillon | 2017 | Yes | Yes | Yes | Yes | Yes | Yes | Yes | Yes | Yes | Yes | Low |
| Chamberland | 2011 | Yes | Yes | No | Yes | Yes | Yes | Yes | Yes | Yes | No | Low |
| Chan | 2011 | No | No | No | Yes | Yes | Yes | Yes | Yes | Yes | No | High |
| Chang | 2008 | Yes | Yes | Yes | Yes | Yes | Yes | Yes | Yes | Yes | No | Low |
| Chen | 2014 | Yes | Yes | No | Yes | No | Yes | Yes | Yes | Yes | Yes | Low |
| Chen | 2012 | No | Yes | No | Yes | Yes | Yes | Yes | Yes | Yes | Yes | Low |
| Chen | 2018 | Yes | Yes | Yes | Yes | Yes | Yes | Yes | Yes | Yes | Yes | Low |
| Chen | 2018 | Yes | Yes | Yes | Yes | Yes | Yes | Yes | Yes | Yes | Yes | Low |
| Chen | 2014 | No | Yes | No | Yes | No | Yes | Yes | Yes | Yes | Yes | Low |
| Chen | 2012 | Yes | Yes | Yes | Yes | Yes | Yes | Yes | Yes | Yes | No | Low |
| Choi | 2008 | Yes | Yes | No | Yes | Yes | Yes | Yes | Yes | Yes | Yes | Low |
| Coetzee | 2017 | No | No | No | Yes | Yes | Yes | Yes | Yes | Yes | Yes | Moderate |
| Colby | 2016 | No | Yes | No | Yes | No | Yes | Yes | Yes | Yes | Yes | Low |
| Collins | 2015 | No | No | No | Yes | Yes | Yes | Yes | Yes | Yes | Yes | High |
| Cuningham | 2014 | Yes | Yes | No | Yes | No | Yes | Yes | Yes | Yes | Yes | Low |
| Dai | 2014 | Yes | Yes | Yes | Yes | Yes | Yes | Yes | Yes | Yes | No | Low |
| Davarpanah | 2017 | No | No | No | No | Yes | Yes | Yes | Yes | Yes | Yes | Moderate |
| de Mendoza | 2005 | No | Yes | No | Yes | Yes | Yes | Yes | Yes | Yes | Yes | Low |
| Dean | 2011 | No | Yes | Yes | Yes | Yes | Yes | Yes | Yes | Yes | Yes | Low |
| Delaugerre | 2018 | No | No | Yes | Yes | No | Yes | Yes | Yes | Yes | No | Low |
| Do | 2017 | Yes | Yes | Yes | Yes | Yes | Yes | Yes | Yes | Yes | Yes | Low |
| Drescher | 2014 | Yes | Yes | No | Yes | Yes | Yes | Yes | Yes | Yes | No | Low |
| Dvali | 2012 | No | Yes | No | Yes | Yes | Yes | Yes | Yes | Yes | No | Low |
| Easterbrook | 2002 | No | No | No | Yes | Yes | Yes | Yes | Yes | Yes | Yes | High |
| Eshleman | 2007 | No | No | No | Yes | Yes | Yes | Yes | Yes | Yes | No | High |
| Eyzaguirre | 2007 | No | No | No | Yes | No | Yes | Yes | Yes | Yes | No | High |
| Fabeni | 2015 | No | Yes | No | Yes | Yes | Yes | Yes | Yes | Yes | No | Low |
| Feng | 2006 | No | No | No | Yes | Yes | Yes | Yes | Yes | Yes | No | High |
| Ferreira | 2012 | Yes | Yes | No | Yes | Yes | Yes | Yes | Yes | Yes | Yes | Low |
| Fox | 2007 | No | No | No | Yes | Yes | Yes | Yes | Yes | Yes | Yes | Moderate |
| Fox | 2006 | No | Yes | No | Yes | Yes | Yes | Yes | Yes | Yes | No | Low |
| Franca | 2018 | No | Yes | No | Yes | Yes | Yes | Yes | Yes | Yes | Yes | Moderate |
| Franzetti | 2012 | Yes | Yes | No | Yes | Yes | Yes | Yes | Yes | Yes | No | Low |
| Frentz | 2014 | Yes | Yes | No | Yes | Yes | Yes | Yes | Yes | Yes | No | Low |
| Gallego | 2003 | No | Yes | No | Yes | Yes | Yes | Yes | Yes | Yes | Yes | Low |
| Garcia-Guerrero | 2006 | No | Yes | No | Yes | Yes | Yes | Yes | Yes | Yes | Yes | Low |
| Garcia-Guerrero | 2004 | No | No | Yes | Yes | Yes | Yes | Yes | Yes | Yes | Yes | Low |
| Garcia-Guerrero | 2002 | No | No | No | Yes | Yes | Yes | Yes | Yes | Yes | Yes | Low |
| Garcia-Morales | 2017 | No | Yes | No | Yes | No | Yes | Yes | Yes | Yes | No | Moderate |
| Gascun | 2012 | Yes | Yes | Yes | Yes | Yes | Yes | Yes | Yes | Yes | Yes | Low |
| Geretti | 2001 | No | Yes | No | Yes | Yes | Yes | Yes | Yes | Yes | No | Low |
| Ghafari | 2017 | No | No | No | No | Yes | Yes | Yes | Yes | Yes | No | High |
| Gianella | 2013 | No | No | No | No | Yes | Yes | Yes | Yes | Yes | No | High |
| Gittens | 2003 | No | No | No | No | Yes | Yes | Yes | Yes | Yes | Yes | High |
| Giuliani | 2009 | No | No | No | No | Yes | Yes | Yes | Yes | Yes | No | High |
| Gonzalez-Domenech | 2018 | No | No | No | Yes | Yes | Yes | Yes | Yes | Yes | No | High |
| Gordon | 2010 | No | No | No | Yes | No | Yes | Yes | Yes | Yes | Yes | Moderate |
| Grgic | 2013 | Yes | Yes | No | Yes | Yes | Yes | Yes | Yes | Yes | Yes | Low |
| Grubb | 2006 | No | Yes | No | Yes | Yes | Yes | Yes | Yes | Yes | No | Low |
| Guanira | 2009 | Yes | Yes | No | Yes | Yes | Yes | Yes | Yes | Yes | No | Low |
| Guimares | 2015 | Yes | Yes | No | Yes | Yes | Yes | Yes | Yes | Yes | Yes | Low |
| Guo | 2017 | No | No | No | No | Yes | Yes | Yes | Yes | Yes | Yes | High |
| Han | 2018 | Yes | Yes | Yes | Yes | Yes | Yes | Yes | Yes | Yes | Yes | Low |
| Hanna | 2003 | No | No | No | No | Yes | Yes | Yes | Yes | Yes | Yes | High |
| Hightow-Weidman | 2011 | Yes | No | No | Yes | Yes | Yes | Yes | Yes | Yes | Yes | Low |
| Hiransuthikul | 2018 | No | No | No | Yes | Yes | Yes | Yes | Yes | Yes | Yes | High |
| Horban | 2002 | No | Yes | Yes | No | Yes | Yes | Yes | Yes | Yes | Yes | Low |
| Hou | 2015 | No | No | No | No | Yes | Yes | Yes | Yes | Yes | Yes | High |
| Hua | 2013 | Yes | Yes | Yes | Yes | Yes | Yes | Yes | Yes | Yes | No | Moderate |
| Huaman | 2011 | Yes | Yes | No | Yes | Yes | Yes | Yes | Yes | Yes | No | Low |
| Hurt | 2009 | No | Yes | No | Yes | No | Yes | Yes | No | Yes | No | High |
| Iarikov | 2010 | Yes | Yes | Yes | Yes | Yes | Yes | Yes | Yes | Yes | No | Moderate |
| Ibe | 2003 | No | No | No | No | Yes | Yes | Yes | Yes | Yes | Yes | High |
| Iqbal | 2009 | No | Yes | Yes | Yes | Yes | Yes | Yes | Yes | Yes | Yes | Low |
| Jayaraman | 2006 | No | Yes | Yes | Yes | Yes | Yes | Yes | Yes | Yes | No | Low |
| Jeong | 2019 | No | No | No | No | Yes | Yes | Yes | Yes | No | No | High |
| Jeulin | 2014 | No | Yes | Yes | Yes | No | Yes | Yes | Yes | Yes | No | Low |
| Jiamsakul | 2014 | Yes | Yes | No | Yes | Yes | Yes | Yes | Yes | Yes | No | Low |
| Jiamsakul | 2015 | No | No | No | No | Yes | Yes | Yes | Yes | Yes | Yes | Moderate |
| Kanizsai | 2010 | No | No | No | No | Yes | Yes | Yes | Yes | Yes | Yes | High |
| Karade | 2016 | No | No | No | No | Yes | Yes | Yes | Yes | Yes | No | High |
| Karlsson | 2012 | Yes | Yes | No | Yes | Yes | Yes | Yes | Yes | Yes | No | Low |
| Khan | 2009 | Yes | Yes | No | Yes | No | Yes | Yes | Yes | Yes | No | Low |
| Kotaki | 2015 | No | No | No | Yes | No | Yes | Yes | Yes | Yes | No | High |
| Lai | 2012 | No | Yes | No | Yes | Yes | Yes | Yes | Yes | Yes | No | Low |
| Lan | 2008 | No | Yes | Yes | Yes | No | Yes | Yes | Yes | Yes | Yes | Low |
| Lapadula | 2008 | No | No | No | Yes | No | Yes | Yes | Yes | Yes | Yes | Moderate |
| Li | 2015 | No | Yes | Yes | Yes | Yes | Yes | Yes | Yes | Yes | No | Low |
| Li | 2015 | Yes | Yes | No | Yes | Yes | Yes | Yes | Yes | Yes | Yes | Low |
| Li | 2015 | No | Yes | Yes | Yes | No | Yes | Yes | Yes | Yes | Yes | Low |
| Li | 2013 | No | No | No | Yes | Yes | Yes | Yes | Yes | Yes | No | High |
| Li | 2013 | No | Yes | No | Yes | Yes | Yes | Yes | Yes | Yes | No | Low |
| Liao | 2010 | Yes | Yes | No | Yes | Yes | Yes | Yes | Yes | Yes | Yes | Low |
| Lindstrom | 2006 | Yes | Yes | No | No | Yes | Yes | Yes | Yes | Yes | Yes | Low |
| Liu | 2012 | No | No | No | Yes | Yes | Yes | Yes | Yes | Yes | Yes | Moderate |
| Lopes Maia | 2006 | No | No | No | Yes | Yes | Yes | Yes | Yes | Yes | Yes | Moderate |
| Lu | 2017 | No | No | No | Yes | Yes | Yes | Yes | Yes | Yes | No | High |
| Lunar | 2013 | Yes | No | Yes | No | Yes | Yes | Yes | Yes | Yes | No | Low |
| Maljkovic | 2003 | No | No | No | Yes | Yes | Yes | Yes | Yes | Yes | No | Moderate |
| Martinez | 1997 | No | No | No | No | Yes | Yes | Yes | Yes | Yes | No | High |
| Masquelier | 2005 | Yes | Yes | No | Yes | No | Yes | Yes | Yes | Yes | No | Low |
| McFaul | 2015 | No | No | No | Yes | Yes | Yes | Yes | Yes | Yes | Yes | Moderate |
| Memarnejadian | 2015 | No | No | No | No | Yes | Yes | Yes | Yes | Yes | Yes | High |
| Mendoza | 2004 | No | No | No | Yes | Yes | Yes | Yes | Yes | Yes | No | High |
| Mendoza | 2016 | No | No | No | Yes | Yes | Yes | Yes | Yes | Yes | Yes | Moderate |
| Metzner | 2010 | No | No | No | No | Yes | Yes | Yes | Yes | Yes | No | High |
| Mezei | 2011 | No | No | No | No | Yes | Yes | Yes | Yes | Yes | Yes | High |
| Mor | 2012 | No | No | No | No | Yes | Yes | Yes | Yes | Yes | No | High |
| Moura | 2015 | No | No | No | Yes | Yes | Yes | Yes | Yes | Yes | Yes | Moderate |
| Moura | 2015 | No | No | No | No | Yes | Yes | Yes | Yes | Yes | No | Moderate |
| Murillo | 2010 | Yes | Yes | Yes | Yes | Yes | Yes | Yes | Yes | Yes | No | Low |
| Ocfemia | 2014 | Yes | Yes | No | Yes | Yes | Yes | Yes | Yes | Yes | Yes | Low |
| Oette | 2012 | Yes | Yes | No | Yes | Yes | Yes | Yes | Yes | Yes | No | Low |
| Oliveira- Filho | 2015 | No | No | No | Yes | Yes | Yes | Yes | Yes | Yes | No | Low |
| Osman | 2013 | No | No | No | No | Yes | Yes | Yes | Yes | Yes | Yes | High |
| Palma | 2007 | Yes | No | Yes | No | Yes | Yes | Yes | Yes | Yes | Yes | Low |
| Pando | 2007 | No | Yes | No | No | Yes | Yes | Yes | Yes | Yes | Yes | Moderate |
| Pando | 2011 | Yes | Yes | Yes | Yes | Yes | Yes | Yes | Yes | Yes | Yes | Low |
| Paraskevis | 2005 | Yes | Yes | No | Yes | Yes | Yes | Yes | Yes | Yes | Yes | Low |
| Parczewski | 2014 | No | Yes | No | Yes | No | Yes | Yes | Yes | Yes | No | Low |
| Parczewski | 2017 | No | No | No | No | No | Yes | Yes | Yes | Yes | No | High |
| Payne | 2008 | Yes | Yes | No | Yes | Yes | Yes | Yes | Yes | Yes | Yes | Low |
| Pillay | 2006 | Yes | Yes | No | Yes | Yes | Yes | Yes | Yes | Yes | Yes | Low |
| Pineda-Pena | 2014 | No | Yes | Yes | Yes | No | Yes | Yes | Yes | Yes | Yes | Low |
| Pontali | 2008 | No | Yes | Yes | Yes | No | Yes | Yes | Yes | Yes | Yes | Low |
| Prellwitz | 2013 | Yes | Yes | Yes | Yes | Yes | Yes | Yes | Yes | Yes | No | Low |
| Prellwitz | 2013 | Yes | Yes | Yes | Yes | Yes | Yes | Yes | Yes | Yes | No | Low |
| Qi | 2013 | No | No | No | No | Yes | Yes | Yes | Yes | Yes | Yes | Moderate |
| Quang | 2015 | Yes | Yes | Yes | Yes | Yes | Yes | Yes | Yes | Yes | No | Low |
| Reuter | 2011 | No | No | No | Yes | Yes | Yes | Yes | Yes | Yes | Yes | Moderate |
| Ristig | 2002 | No | No | No | No | Yes | Yes | Yes | Yes | Yes | No | High |
| Riva | 2010 | Yes | No | Yes | Yes | Yes | Yes | Yes | Yes | Yes | Yes | Low |
| Rossetti | 2018 | Yes | Yes | No | Yes | Yes | Yes | Yes | Yes | Yes | Yes | Low |
| Roudinskii | 2004 | No | No | No | No | Yes | Yes | Yes | Yes | Yes | Yes | High |
| Sagir | 2007 | No | Yes | No | Yes | Yes | Yes | Yes | Yes | Yes | No | Low |
| Salama | 2009 | No | Yes | No | Yes | Yes | Yes | Yes | Yes | Yes | Yes | Low |
| Salama | 2011 | No | Yes | Yes | Yes | Yes | Yes | Yes | Yes | Yes | No | Low |
| Sampathkumar | 2013 | No | No | No | Yes | Yes | Yes | Yes | Yes | Yes | No | Moderate |
| Sanabani | 2011 | No | No | No | Yes | Yes | Yes | Yes | Yes | Yes | Yes | High |
| Sapozhnikov | 2017 | No | Yes | No | Yes | No | Yes | Yes | Yes | Yes | Yes | High |
| Shaw | 2012 | No | No | No | No | Yes | No | No | No | Yes | No | High |
| Shen | 2014 | No | Yes | No | No | Yes | Yes | Yes | Yes | Yes | Yes | Moderate |
| Shih | 2015 | No | No | No | Yes | Yes | Yes | Yes | Yes | Yes | No | Low |
| Sirivichayakul | 2016 | No | Yes | No | Yes | Yes | Yes | Yes | Yes | Yes | No | Low |
| Skoura | 2011 | No | No | No | No | Yes | Yes | Yes | Yes | Yes | Yes | High |
| Spread programme | 2008 | Yes | Yes | No | Yes | Yes | Yes | Yes | Yes | Yes | No | Low |
| Sprinz | 2009 | No | Yes | No | No | Yes | Yes | Yes | Yes | Yes | Yes | Moderate |
| Ssemwanga | 2012 | No | No | No | Yes | Yes | Yes | Yes | Yes | Yes | No | High |
| Stone | 2002 | No | No | No | No | Yes | Yes | Yes | Yes | Yes | Yes | High |
| Sullivan | 2013 | No | No | No | No | Yes | Yes | No | Yes | Yes | Yes | High |
| Sullivan | 2002 | No | No | No | No | Yes | Yes | Yes | Yes | Yes | Yes | High |
| Tamalet | 2017 | No | No | No | No | Yes | Yes | Yes | Yes | Yes | Yes | High |
| Taniguchi | 2012 | No | No | No | No | Yes | Yes | Yes | Yes | Yes | Yes | Moderate |
| Tanuma | 2014 | Yes | Yes | Yes | Yes | Yes | No | Yes | Yes | Yes | Yes | Low |
| Teixeria | 2006 | Yes | Yes | Yes | Yes | Yes | Yes | Yes | Yes | Yes | Yes | Low |
| Tetrault | 2013 | No | No | No | Yes | Yes | Yes | Yes | Yes | Yes | No | Low |
| Thomson | 2007 | No | No | No | No | Yes | Yes | Yes | Yes | Yes | Yes | High |
| Todesco | 2017 | No | No | No | No | Yes | Yes | Yes | Yes | Yes | Yes | High |
| Torian | 2013 | No | No | No | No | Yes | No | No | Yes | Yes | Yes | High |
| Tossonian | 2009 | No | Yes | No | Yes | Yes | Yes | Yes | Yes | Yes | Yes | Low |
| Tran | 2012 | No | Yes | No | Yes | Yes | Yes | Yes | Yes | Yes | No | Low |
| Truong | 2010 | Yes | Yes | No | Yes | Yes | Yes | Yes | Yes | Yes | Yes | Low |
| Tsai | 2018 | No | No | No | Yes | Yes | Yes | Yes | Yes | Yes | No | Moderate |
| Tupinambas | 2013 | No | No | No | No | Yes | Yes | Yes | Yes | Yes | No | High |
| Turner | 2012 | No | No | No | No | Yes | No | Yes | Yes | Yes | Yes | High |
| Turner | 2010 | No | No | No | No | Yes | No | Yes | Yes | Yes | Yes | High |
| UK Collaborative Group on Monitoring the Transmission of HIV Drug Resistance | 2001 | No | No | No | No | No | Yes | Yes | Yes | Yes | No | High |
| Vercauteren | 2008 | Yes | Yes | Yes | Yes | Yes | Yes | Yes | Yes | Yes | Yes | Low |
| Viciana | 2015 | No | No | No | No | Yes | Yes | Yes | Yes | Yes | No | High |
| Violin | 2004 | No | No | No | No | Yes | Yes | No | Yes | Yes | Yes | High |
| Violin | 2004 | No | Yes | No | Yes | Yes | Yes | Yes | Yes | Yes | No | Low |
| Weinstock | 2004 | Yes | Yes | No | Yes | Yes | Yes | Yes | Yes | Yes | No | Low |
| Weng | 2014 | No | Yes | Yes | Yes | Yes | Yes | Yes | Yes | Yes | Yes | Low |
| Wheeler | 2010 | Yes | Yes | No | Yes | Yes | Yes | Yes | Yes | Yes | Yes | Low |
| Wong | 2015 | No | No | No | No | Yes | Yes | No | Yes | Yes | Yes | High |
| Xu | 2018 | No | Yes | No | Yes | Yes | Yes | Yes | Yes | Yes | Yes | Low |
| Yan | 2015 | No | Yes | No | No | Yes | Yes | Yes | Yes | Yes | Yes | Moderate |
| Yang | 2015 | Yes | Yes | No | Yes | Yes | Yes | Yes | Yes | Yes | No | Low |
| Yang | 2012 | Yes | Yes | No | Yes | Yes | Yes | Yes | Yes | Yes | Yes | Low |
| Yang | 2012 | No | No | No | Yes | Yes | Yes | Yes | Yes | Yes | Yes | Moderate |
| Ye | 2012 | No | Yes | No | No | Yes | Yes | Yes | Yes | Yes | Yes | High |
| Ye | 2011 | No | No | No | No | Yes | No | No | Yes | Yes | Yes | High |
| Yebra | 2011 | No | No | No | No | Yes | Yes | Yes | Yes | Yes | Yes | Moderate |
| Yebra | 2013 | Yes | Yes | No | Yes | Yes | Yes | Yes | Yes | Yes | Yes | Low |
| Yebra | 2013 | Yes | Yes | No | Yes | Yes | Yes | Yes | Yes | Yes | Yes | Low |
| Youmans | 2011 | Yes | Yes | No | Yes | Yes | Yes | Yes | Yes | Yes | Yes | Low |
| Yu | 2009 | No | No | No | Yes | Yes | Yes | Yes | Yes | Yes | Yes | High |
| Zarandia | 2006 | No | No | No | No | Yes | Yes | Yes | Yes | Yes | No | High |
| Zhang | 2015 | No | No | No | No | Yes | Yes | Yes | Yes | Yes | No | High |
| Zhang | 2007 | No | No | No | Yes | Yes | Yes | Yes | Yes | Yes | Yes | Moderate |
| Zhang | 2017 | No | Yes | No | Yes | Yes | Yes | Yes | Yes | Yes | Yes | Moderate |
| Zhang | 2015 | No | No | No | No | Yes | No | Yes | Yes | Yes | Yes | High |
| Zhao | 2015 | Yes | Yes | Yes | Yes | Yes | Yes | Yes | Yes | Yes | Yes | Low |
| Zhao | 2012 | No | No | No | Yes | Yes | Yes | Yes | Yes | Yes | Yes | High |
| Zhao | 2011 | Yes | Yes | No | Yes | Yes | Yes | Yes | Yes | Yes | Yes | Low |
| Zhing | 2003 | No | No | No | Yes | Yes | Yes | Yes | Yes | Yes | Yes | Moderate |
| Zuckerman | 2019 | Yes | Yes | Yes | Yes | Yes | Yes | Yes | Yes | Yes | Yes | Low |

**Table 5: Prevalence of PDR by region**

| **Key population** |  | **Resistance type** | | | | | | | | | | | |
| --- | --- | --- | --- | --- | --- | --- | --- | --- | --- | --- | --- | --- | --- |
|  |  | **Any** | | | **NNRTI** | | | **NRTI** | | | **PI** | | |
|  |  | **Med** | **Min** | **Max** | **Med** | **Min** | **Max** | **Med** | **Min** | **Max** | **Med** | **Min** | **Max** |
| **SW** | **Africa** | 19.11 | 2.50 | 35.71 | .01 | .01 | .02 | 10.72 | .02 | 21.43 | 9.83 | .02 | 19.64 |
|  | **America** | 12.80 | 1.56 | 100.00 | 9.58 | 1.56 | 80.00 | 8.77 | 1.56 | 60.00 | 7.16 | 1.56 | 60.00 |
|  | **Europe** | 25.00 | 25.00 | 25.00 | 25.00 | 25.00 | 25.00 | 25.00 | 25.00 | 25.00 | 25.00 | 25.00 | 25.00 |
|  | **South East Asia** | 8.33 | 8.33 | 8.33 | 8.33 | 8.33 | 8.33 | .17 | .17 | .17 | .17 | .17 | .17 |
|  | **Western Pacific** | 2.71 | .15 | 5.26 | 2.71 | .15 | 5.26 | .08 | .02 | .15 | .08 | .02 | .15 |
| **MSM** | **America** | 15.13 | .03 | 100.00 | 6.20 | .02 | 18.18 | 5.41 | .01 | 50.00 | 4.17 | .01 | 25.00 |
|  | **Europe** | 10.90 | 3.03 | 74.07 | 4.14 | .00 | 46.67 | 5.16 | .93 | 66.67 | 1.72 | .00 | 40.74 |
|  | **South East Asia** | 7.08 | 4.26 | 100.00 | 2.78 | .01 | 71.43 | 5.56 | .02 | 28.57 | 3.77 | .01 | 71.43 |
|  | **Western Pacific** | 4.74 | 1.00 | 62.50 | 2.18 | .01 | 28.57 | 1.02 | .00 | 55.00 | 2.29 | .00 | 50.00 |
| **Prisoners** | **America** | 23.68 | 22.86 | 23.68 | 13.16 | 13.16 | 16.19 | 5.26 | 5.26 | 8.57 | 5.26 | 2.86 | 5.26 |
|  | **Europe** | 11.63 | 7.78 | 13.83 | 6.98 | 4.44 | 8.51 | 5.32 | 2.22 | 6.98 | 2.13 | 1.11 | 6.98 |
|  | **Western Pacific** | 14.29 | 14.29 | 14.29 | 14.29 | 14.29 | 14.29 | 4.76 | 4.76 | 4.76 | .06 | .06 | .06 |
| **PWID** | **Africa** | 13.79 | 13.79 | 13.79 | 1.72 | 1.72 | 1.72 | 13.79 | 13.79 | 13.79 | .01 | .01 | .01 |
|  | **America** | 18.34 | .01 | 100.00 | 4.55 | .01 | 66.67 | 6.70 | .00 | 66.67 | 5.43 | .00 | 86.15 |
|  | **Eastern Mediterranean** | 15.00 | 15.00 | 15.00 | 7.50 | 5.00 | 10.00 | 7.50 | 5.00 | 10.00 | 2.51 | .02 | 5.00 |
|  | **Europe** | 6.25 | .03 | 57.83 | 3.85 | .00 | 25.00 | 3.70 | .00 | 57.83 | 2.45 | .00 | 25.00 |
|  | **South East Asia** | 25.00 | 7.27 | 42.11 | 21.05 | 1.82 | 25.00 | 15.79 | 3.64 | 25.00 | 15.79 | 1.82 | 25.00 |
|  | **Western Pacific** | 4.57 | .00 | 50.00 | 1.11 | .00 | 50.00 | 2.31 | .00 | 30.30 | .68 | .00 | 50.00 |

*SW: sex workers; MSM: men who have sex with men; PWID: people who inject drugs; NNRTI: non-nucleoside reverse transcriptase inhibitor; NNRTI: nucleoside revere transcriptase inhibitor; PI: protease inhibitor; Med: median; min: Minimum; Max: maximum. Median effect sizes computed

**Table 6: Prevalence of PDR by income level**

| **Key population** | **Income level** | **Resistance type** | | | | | | | | | | | |
| --- | --- | --- | --- | --- | --- | --- | --- | --- | --- | --- | --- | --- | --- |
|  |  | **Any** | | | **NNRTI** | | | **NRTI** | | | **PI** | | |
|  |  | **Med** | **Min** | **Max** | **Med** | **Med** | **Min** | **Max** | **Med** | **Med** | **Min** | **Max** | **Max** |
| **SW** | **Low** | 19.11 | 2.50 | 35.71 | .01 | .01 | .02 | 10.72 | .02 | 21.43 | 9.83 | .02 | 19.64 |
|  | **Low Middle** | 8.33 | 5.26 | 25.00 | 8.33 | 5.26 | 25.00 | .17 | .02 | 25.00 | .17 | .02 | 25.00 |
|  | **Upper Middle** | 6.25 | .15 | 100.00 | 6.25 | .15 | 80.00 | 6.25 | .15 | 60.00 | 6.25 | .15 | 60.00 |
| **MSM** | **High** | 10.99 | 3.03 | 74.07 | 4.75 | .00 | 46.67 | 5.39 | .01 | 66.67 | 1.92 | .00 | 40.74 |
|  | **Low** | 6.25 | 6.25 | 6.25 | 6.25 | 6.25 | 6.25 | 6.25 | 6.25 | 6.25 | 6.25 | 6.25 | 6.25 |
|  | **Low Middle** | 6.25 | .03 | 33.33 | 6.25 | .03 | 33.33 | 6.25 | .03 | 11.11 | 6.25 | .03 | 11.11 |
|  | **Mixed*** | 4.27 | 4.27 | 4.27 | 2.56 | 2.56 | 2.56 | .85 | .85 | .85 | .85 | .85 | .85 |
|  | **Upper Middle** | 8.33 | 1.00 | 100.00 | 3.20 | .01 | 71.43 | 2.11 | .00 | 55.00 | 3.70 | .00 | 71.43 |
| **Prisoners** | **High** | 12.73 | 7.78 | 22.86 | 7.74 | 4.44 | 16.19 | 6.15 | 2.22 | 8.57 | 2.49 | 1.11 | 6.98 |
|  | **Upper Middle** | 23.68 | 14.29 | 23.68 | 13.16 | 13.16 | 14.29 | 5.26 | 4.76 | 5.26 | 5.26 | .06 | 5.26 |
| **PWID** | **High** | 8.43 | .01 | 57.83 | 4.61 | .00 | 27.75 | 4.91 | .00 | 57.83 | 1.89 | .00 | 25.00 |
|  | **Low** | 25.00 | 25.00 | 25.00 | 25.00 | 25.00 | 25.00 | 25.00 | 25.00 | 25.00 | 25.00 | 25.00 | 25.00 |
|  | **Low Middle** | 6.90 | .00 | 42.11 | 1.72 | .00 | 21.05 | 3.64 | .00 | 15.79 | 1.01 | .00 | 15.79 |
|  | **Upper Middle** | 15.00 | .05 | 100.00 | 2.78 | .00 | 66.67 | 5.00 | .00 | 66.67 | 4.62 | .00 | 86.15 |

* includes countries from more than one category; *SW: sex workers; MSM: men who have sex with men; PWID: people who inject drugs; NNRTI: non-nucleoside reverse transcriptase inhibitor; NNRTI: nucleoside revere transcriptase inhibitor; PI: protease inhibitor; Med: median; min: Minimum; Max: maximum. Median effect sizes computed

Reference List:

1. Acevedo W, Gallardo AM, Galaz J, Afani A, Cortes E. [Detection of primary antiretroviral resistance in Chilean patients recently infected with human immunodeficiency virus (HIV)]. Rev Med Chil. 2007;135(11):1406-13.

2. Alexander CS, Dong W, Schechter MT, O'Shaughnessy MV, Strathdee SA, Mo T, et al. Prevalence of primary HIV drug resistance among seroconverters during an explosive outbreak of HIV infection among injecting drug users. AIDS. 1999;13(8):981-5.

3. Alexiev I, Shankar A, Dimitrova R, Gancheva A, Kostadinova A, Teoharov P, et al. Origin and spread of HIV-1 in persons who inject drugs in Bulgaria. Infect Genet Evol. 2016;46:269-78.

4. Alexiev I, Shankar A, Wensing AM, Beshkov D, Elenkov I, Stoycheva M, et al. Low HIV-1 transmitted drug resistance in Bulgaria against a background of high clade diversity. J Antimicrob Chemother. 2015;70(6):1874-80.

5. Allison L, Hightow W, Lisa B, John W, Craig M, Bill G. Substantial multiclass transmitted drug resistance and drug-relevant polymorphisms among treatment-naïve behaviorally HIV-infected youth. AIDS Patient Care and STDs. 2012;26(4):193-6.

6. Alpsar D, Agacfidan A, Lubke N, Verheyen J, Eraksoy H, Cagatay A, et al. Molecular epidemiology of HIV in a cohort of men having sex with men from Istanbul. Med Microbiol Immunol. 2013;202(3):251-5.

7. Ananworanich J, Phanuphak N, de Souza M, Paris R, Arroyo M, Trichavaroj R, et al. Incidence and characterization of acute HIV-1 infection in a high-risk Thai population. J Acquir Immune Defic Syndr. 2008;49(2):151-5.

8. Ananworanich J, Sirivichayakul S, Pinyakorn S, Crowell TA, Trichavaroj R, Weerayingyong J, et al. High prevalence of transmitted drug resistance in acute HIV-infected Thai men who have sex with men. J Acquir Immune Defic Syndr. 2015;68(4):481-5.

9. Andersson E, Nordquist A, Esbjornsson J, Flamholc L, Gisslen M, Hejdeman B, et al. Increase in transmitted drug resistance in migrants from sub-Saharan Africa diagnosed with HIV-1 in Sweden. AIDS. 2018;32(7):877-84.

10. Andreani G, Espada C, Ceballos A, Ambrosioni J, Petroni A, Pugliese D, et al. Detection of HIV-1 dual infections in highly exposed treated patients. Virology journal. 2011;8(1):392.

11. Ariffin TA, Mohamad S, Yusuf WN, Shueb RH. Antiretroviral drug resistance and HIV-1 subtypes among treatment-naive prisoners in Kelantan, Malaysia. J Infect Dev Ctries. 2014;8(8):1063-7.

12. Audelin AM, Gerstoft J, Obel N, Mathiesen L, Laursen A, Pedersen C, et al. Molecular phylogenetics of transmitted drug resistance in newly diagnosed HIV Type 1 individuals in Denmark: a nation-wide study. AIDS Res Hum Retroviruses. 2011;27(12):1283-90.

13. Avila-Rios S, Garcia-Morales C, Matias-Florentino M, Romero-Mora KA, Tapia-Trejo D, Quiroz-Morales VS, et al. Pretreatment HIV-drug resistance in Mexico and its impact on the effectiveness of first-line antiretroviral therapy: a nationally representative 2015 WHO survey. The lancet HIV. 2016;3(12):e579-e91.

14. Avila-Rios S, Mejia-Villatoro CR, Garcia-Morales C, Soto-Nava M, Escobar I, Mendizabal R, et al. Prevalence and patterns of HIV transmitted drug resistance in Guatemala. Revista panamericana de salud publica = Pan American journal of public health. 2011;30(6):641-8.

15. Babic DZ, Zelnikar M, Seme K, Vandamme AM, Snoeck J, Tomazic J, et al. Prevalence of antiretroviral drug resistance mutations and HIV-1 non-B subtypes in newly diagnosed drug-naive patients in Slovenia, 2000-2004. Virus Res. 2006;118(1-2):156-63.

16. Bannister WP, Cozzi-Lepri A, Clotet B, Mocroft A, Kjaer J, Reiss P, et al. Transmitted drug resistant HIV-1 and association with virologic and CD4 cell count response to combination antiretroviral therapy in the EuroSIDA Study. J Acquir Immune Defic Syndr. 2008;48(3):324-33.

17. Bermúdez-Aza EH, Kerr LRFS, Kendall C, Pinho AA, de Mello MB, Mota RS, et al. Antiretroviral drug resistance in a respondent-driven sample of HIV-infected men who have sex with men in Brazil. JAIDS Journal of Acquired Immune Deficiency Syndromes. 2011;57:S186-S92.

18. Bezemer D, Jurriaans S, Prins M, van der Hoek L, Prins JM, de Wolf F, et al. Declining trend in transmission of drug-resistant HIV-1 in Amsterdam. AIDS. 2004;18(11):1571-7.

19. Bhusal N, Sutthent R, Horthongkham N, Athipanyasilp N, Kantakamalakul W. Prevalence of HIV-1 Subtypes and Antiretroviral Drug Resistance Mutations in Nepal. Curr HIV Res. 2016;14(6):517-24.

20. Bontell I, Cuong do D, Agneskog E, Diwan V, Larsson M, Sonnerborg A. Transmitted drug resistance and phylogenetic analysis of HIV CRF01_AE in Northern Vietnam. Infect Genet Evol. 2012;12(2):448-52.

21. Bonura F, Tramuto F, Vitale F, Perna AM, Viviano E, Romano N, et al. Transmission of drug-resistant HIV type 1 strains in HAART-naive patients: a 5-year retrospective study in Sicily, Italy. AIDS Res Hum Retroviruses. 2010;26(9):961-5.

22. Booth CL, Garcia-Diaz AM, Youle MS, Johnson MA, Phillips A, Geretti AM. Prevalence and predictors of antiretroviral drug resistance in newly diagnosed HIV-1 infection. J Antimicrob Chemother. 2007;59(3):517-24.

23. Brindeiro RM, Diaz RS, Sabino EC, Morgado MG, Pires IL, Brigido L, et al. Brazilian Network for HIV Drug Resistance Surveillance (HIV-BResNet): a survey of chronically infected individuals. Aids. 2003;17(7):1063-9.

24. Briones C, Perez-Olmeda M, Rodriguez C, del Romero J, Hertogs K, Soriano V. Primary genotypic and phenotypic HIV-1 drug resistance in recent seroconverters in Madrid. J Acquir Immune Defic Syndr. 2001;26(2):145-50.

25. Brooks JI, Niznick H, Ofner M, Merks H, Angel JB. Local phylogenetic analysis identifies distinct trends in transmitted HIV drug resistance: implications for public health interventions. BMC Infect Dis. 2013;13:509.

26. Buchacz K, Young B, Palella FJ, Jr., Armon C, Brooks JT, investigators HIVOS, et al. Trends in use of genotypic resistance testing and frequency of major drug resistance among antiretroviral-naive persons in the HIV Outpatient Study, 1999-2011. J Antimicrob Chemother. 2015;70(8):2337-46.

27. Budambula V, Musumba FO, Webale MK, Kahiga TM, Ongecha-Owuor F, Kiarie JN, et al. HIV-1 protease inhibitor drug resistance in Kenyan antiretroviral treatment-naive and -experienced injection drug users and non-drug users. AIDS Res Ther. 2015;12:27.

28. Burchell A, Bayoumi A, Major C, Gardner S, Taylor D, Rachlis A, et al. P3-S3. 12 Transmitted HIV drug resistance mutations in Ontario, Canada, 2002–2009. Sex Transm Infect. 2011;87(Suppl 1):A289-A.

29. Cardoso LP, Queiroz BB, Stefani MM. HIV-1 pol phylogenetic diversity and antiretroviral resistance mutations in treatment naive patients from Central West Brazil. J Clin Virol. 2009;46(2):134-9.

30. Carobene M, Bolcic F, Farias MS, Quarleri J, Avila MM. HIV, HBV, and HCV molecular epidemiology among trans (transvestites, transsexuals, and transgender) sex workers in Argentina. J Med Virol. 2014;86(1):64-70.

31. Carvalho BC, Cardoso LP, Damasceno S, Stefani MM. Moderate prevalence of transmitted drug resistance and interiorization of HIV type 1 subtype C in the inland North State of Tocantins, Brazil. AIDS Res Hum Retroviruses. 2011;27(10):1081-7.

32. Cavalcanti AM, Brito AM, Salustiano DM, Lima KO, Silva SP, Diaz RS, et al. Primary resistance of HIV to antiretrovirals among individuals recently diagnosed at voluntary counselling and testing centres in the metropolitan region of Recife, Pernambuco. Mem Inst Oswaldo Cruz. 2012;107(4):450-7.

33. Cecchini D, Castillo S, Vecchio C, Sandoval C, Cabral L, Rodriguez Iantorno P, et al. [Primary HIV resistance in Buenos Aires metropolitan area]. Medicina (B Aires). 2015;75(3):163-8.

34. Chaillon A, Nakazawa M, Wertheim JO, Little SJ, Smith DM, Mehta SR, et al. No Substantial Evidence for Sexual Transmission of Minority HIV Drug Resistance Mutations in Men Who Have Sex with Men. J Virol. 2017;91(21).

35. Chamberland A, Diabaté S, Sylla M, Anagounou S, Geraldo N, Zannou DM, et al. Transmission of HIV-1 drug resistance in Benin could jeopardise future treatment options. Sex Transm Infect. 2012;88(3):179-83.

36. Chan PA, Tashima K, Cartwright CP, Gillani FS, Mintz O, Zeller K, et al. Short communication: Transmitted drug resistance and molecular epidemiology in antiretroviral naive HIV type 1-infected patients in Rhode Island. AIDS Res Hum Retroviruses. 2011;27(3):275-81.

37. Chang SY, Chen MY, Lee CN, Sun HY, Ko W, Chang SF, et al. Trends of antiretroviral drug resistance in treatment-naive patients with human immunodeficiency virus type 1 infection in Taiwan. J Antimicrob Chemother. 2008;61(3):689-93.

38. Chen I, Cummings V, Wang L, Connor MB, Marzinke MA, Fields SD, et al. Antiretroviral drug resistance among HIV-infected black men who have sex with men in the US. CROI; 3-6 March, 2014; Boston, USA2014. p. 285.

39. Chen M, Jia MH, Ma YL, Luo HB, Chen HC, Yang CJ, et al. The changing HIV-1 genetic characteristics and transmitted drug resistance among recently infected population in Yunnan, China. Epidemiol Infect. 2018;146(6):775-81.

40. Chen M, Ma Y, Chen H, Dai J, Dong L, Yang C, et al. HIV-1 genetic transmission networks among men who have sex with men in Kunming, China. PLoS One. 2018;13(4):e0196548.

41. Chen M, Ma Y, Duan S, Xing H, Yao S, Su Y, et al. Genetic diversity and drug resistance among newly diagnosed and antiretroviral treatment-naive HIV-infected individuals in western Yunnan: a hot area of viral recombination in China. BMC Infect Dis. 2012;12:382.

42. Chen M, Ma Y, Su Y, Yang L, Zhang R, Yang C, et al. HIV-1 genetic characteristics and transmitted drug resistance among men who have sex with men in Kunming, China. PloS one. 2014;9(1).

43. Chen S, Cai W, He J, Vidal N, Lai C, Guo W, et al. Molecular epidemiology of human immunodeficiency virus type 1 in Guangdong province of southern China. PLoS One. 2012;7(11):e48747.

44. Choi JY, Kim EJ, Park YK, Lee JS, Kim SS. National survey for drug-resistant variants in newly diagnosed antiretroviral drug-naive patients with HIV/AIDS in South Korea: 1999-2005. J Acquir Immune Defic Syndr. 2008;49(3):237-42.

45. Coetzee J, Hunt G, Jaffer M, Otwombe K, Scott L, Bongwe A, et al. HIV-1 viraemia and drug resistance amongst female sex workers in Soweto, South Africa: A cross sectional study. PLoS One. 2017;12(12):e0188606.

46. Colby D, Phanuphak N, Sirivichayakul S, Prueksakaew P, Saengtawan P, Trichavaroj R, et al. HIV transmitted drug resistance declined from 2009 to 2014 among acutely infected MSM in Bangkok, Thailand. Journal of the International Aids Society. 2015;18.

47. Colby DJ, Crowell TA, Sirivichayakul S, Pinyakorn S, Kroon E, Benjapornpong K, et al. Declining trend in transmitted drug resistance detected in a prospective cohort study of acute HIV infection in Bangkok, Thailand. J Int AIDS Soc. 2016;19(1):20966.

48. Collins-Fairclough AM, Dennis AM, Nelson JA, Weir SS, Figueroa JP. HIV Drug Resistance Surveillance Among Jamaican Men Who Have Sex with Men Should Be Prioritized for Reducing HIV Transmission. AIDS Res Hum Retroviruses. 2015;31(8):841-4.

49. Cozzi L, D'Arminio M, C F. Risk of failure in patients with 215 HIV-1 revertants starting their first thymidine analog-containing highly active antiretroviral therapy. AIDS. 2004;18(2):227-35.

50. Dai L, Li N, Wei F, Li J, Liu Y, Xia W, et al. Transmitted antiretroviral drug resistance in the men who have sex with men HIV patient cohort, Beijing, China, 2008-2011. Viral Immunol. 2014;27(8):392-7.

51. Dai L, Mahajan S, Sykes D, Nair B, Schwartz S. Quantitative transmitted drug resistance (TDR) variation in acute/recently infected men who have sex with men (MSM) Chinese HIV patient cohort. J Antivir Antiretrovir. 2013;6:13-21.

52. Davarpanah MA, Motazedian N, Joulaei H, Aghasadeghi MR, Faramarzi H, Aghah E. Comparison of antiretroviral drug resistance among treatment-naive and treated HIV-infected individuals in Shiraz, Iran. Arch Virol. 2018;163(1):99-104.

53. De Gascun CF, Waters A, Regan C, O'Halloran J, Farrell G, Coughlan S, et al. Documented prevalence of HIV type 1 antiretroviral transmitted drug resistance in Ireland from 2004 to 2008. AIDS Res Hum Retroviruses. 2012;28(3):276-81.

54. de Medeiros LB, Lacerda HR, Cavalcanti AM, de Albuquerque Mde F. Primary resistance of human immunodeficiency virus type 1 in a reference center in Recife, Pernambuco, Brazil. Mem Inst Oswaldo Cruz. 2006;101(8):845-9.

55. de Mendoza C, Rodriguez C, Corral A, del Romero J, Gallego O, Soriano V. Evidence for differences in the sexual transmission efficiency of HIV strains with distinct drug resistance genotypes. Clin Infect Dis. 2004;39(8):1231-8.

56. de Mendoza C, Rodriguez C, Eiros JM, Colomina J, Garcia F, Leiva P, et al. Antiretroviral recommendations may influence the rate of transmission of drug-resistant HIV type 1. Clin Infect Dis. 2005;41(2):227-32.

57. Dean J, Ta Thi TH, Dunford L, Carr MJ, Nguyen LT, Coughlan S, et al. Prevalence of HIV type 1 antiretroviral drug resistance mutations in Vietnam: a multicenter study. AIDS Res Hum Retroviruses. 2011;27(7):797-801.

58. Delaugerre C, Rodriguez C, Capitant C, Nere ML, Mercier-Darty M, Carette D, et al. Drug resistance among patients who acquired HIV infection in a preexposure prophylaxis trial. AIDS. 2018;32(16):2353-61.

59. Do HT, Nguyen DT, Nguyen LAT, Do DH, Le HX, Trinh XMT, et al. An Alarmingly High Proportion of HIV-1 Isolates Carrying Mutations Corresponding to Resistance to Antiretroviral Drugs among HIV-Positive High-Risk Groups in Central Vietnam: a Substudy of the National Sentinel Survey. Jpn J Infect Dis. 2017;70(6):621-7.

60. Drescher SM, von Wyl V, Yang W-L, Böni J, Yerly S, Shah C, et al. Treatment-naive individuals are the major source of transmitted HIV-1 drug resistance in men who have sex with men in the Swiss HIV Cohort Study. Clinical infectious diseases. 2014;58(2):285-94.

61. Dvali N, Parker MM, Chkhartishvili N, Sharvadze L, Gochitashvili N, Abutidze A, et al. Characterization of HIV‐1 subtypes and drug resistance mutations among individuals infected with HIV in Georgia. Journal of medical virology. 2012;84(7):1002-8.

62. Easterbrook PJ, Hertogs K, Waters A, Wills B, Gazzard BG, Larder B. Low prevalence of antiretroviral drug resistance among HIV-1 seroconverters in London, 1984-1991. J Infect. 2002;44(2):88-91.

63. Eshleman SH, Husnik M, Hudelson S, Donnell D, Huang Y, Huang W, et al. Antiretroviral drug resistance, HIV-1 tropism, and HIV-1 subtype among men who have sex with men with recent HIV-1 infection. AIDS. 2007;21(9):1165-74.

64. Eyzaguirre L, Brouwer K, Nadai Y, Patterson T, Ramos R, Cruz MF, et al. First Molecular Surveillance Report of HIV Type 1 in Injecting Drug Users and Female Sex Workers along the US–Mexico Border. AIDS research and human retroviruses. 2007;23(2):331-4.

65. Fabeni L, Alteri C, Orchi N, Gori C, Bertoli A, Forbici F, et al. Recent Transmission Clustering of HIV-1 C and CRF17_BF Strains Characterized by NNRTI-Related Mutations among Newly Diagnosed Men in Central Italy. PLoS One. 2015;10(8):e0135325.

66. Feng LG, Wang MJ, Han M, Ding XB, Jiang Y. [Drug resistance among recent HIV-1 infected men who have sex with men in Chongqing municipality of China]. Zhonghua Liu Xing Bing Xue Za Zhi. 2008;29(5):455-8.

67. Ferreira JL, Rodrigues R, Lanca AM, de Almeida VC, Rocha SQ, Ragazzo TG, et al. Transmitted Drug Resistance among People Living with HIV/Aids at Major Cities of Sao Paulo State, Brazil. Adv Virol. 2013;2013:878237.

68. Fox J, Dustan S, McClure M, Weber J, Fidler S. Transmitted drug-resistant HIV-1 in primary HIV-1 infection; incidence, evolution and impact on response to antiretroviral therapy. HIV Med. 2006;7(7):477-83.

69. Fox J, Hill S, Kaye S, Dustan S, McClure M, Fidler S, et al. Prevalence of primary genotypic resistance in a UK centre: comparison of primary HIV-1 and newly diagnosed treatment-naive individuals. Aids. 2007;21(2):237-9.

70. Franca D, Del-Rios NHA, Carneiro M, Guimaraes RA, Caetano KAA, Reis M, et al. HIV-1 infection among crack cocaine users in a region far from the epicenter of the HIV epidemic in Brazil: Prevalence and molecular characteristics. PLoS One. 2018;13(7):e0199606.

71. Franzetti M, Lai A, Simonetti FR, Bozzi G, De Luca A, Micheli V, et al. High burden of transmitted HIV-1 drug resistance in Italian patients carrying F1 subtype. J Antimicrob Chemother. 2012;67(5):1250-3.

72. Frentz D, van de Vijver D, Abecasis A, Albert J, Hamouda O, Jørgensen L, et al. Patterns of transmitted HIV drug resistance in Europe vary by risk group. PloS one. 2014;9(4).

73. García G, J C, Herrero M, Vera R, Castellano C, J C, et al. Primary HIV drug resistance in a prison population. REPRICOVA-2 Study. Enfermedades Infecciosas y Microbiologia Clinica. 2004;22(1):29-31.

74. Garcia G, J M, J C, ez C, M I, Gonzalez M. Mutations of resistance of HIV-1 in previously untreated patients of penitentiary centers of the autonomous community of Valencia, Spain. REPRICOVA study. Medicina Clinica. 2002;118(7):247-50.

75. Garcia G, Saiz De La H, Sanchez P, Garcia B, Ruiz R. Prevalence of HIV-1 drug resistance mutations among Spanish prison inmates. European Journal of Clinical Microbiology and Infectious Diseases. 2006;25(11):695-701.

76. Garcia-Morales C, Tapia-Trejo D, Quiroz-Morales VS, Navarro-Alvarez S, Barrera-Arellano CA, Casillas-Rodriguez J, et al. HIV pretreatment drug resistance trends in three geographic areas of Mexico. J Antimicrob Chemother. 2017;72(11):3149-58.

77. Geretti AM, Smith M, Osner N, O'Shea S, Chrystie I, Easterbrook P, et al. Prevalence of antiretroviral resistance in a South London cohort of treatment-naive HIV-1-infected patients. AIDS. 2001;15(8):1082-4.

78. Ghafari S, Memarnejadian A, Samarbaf-Zadeh A, Mostafavi E, Makvandi M, Salmanzadeh S, et al. Prevalence of HIV-1 transmitted drug resistance in recently infected, treatment-naive persons in the Southwest of Iran, 2014-2015. Arch Virol. 2017;162(9):2737-45.

79. Gianella S, Morris SR, Anderson C, Spina CA, Vargas MV, Young JA, et al. Herpes viruses and HIV-1 drug resistance mutations influence the virologic and immunologic milieu of the male genital tract. AIDS. 2013;27(1):39-47.

80. Gittens MV, Roth WW, Roach T, Stringer HG, Jr., Pieniazek D, Bond VC, et al. The molecular epidemiology and drug resistance determination of HIV type 1 subtype B infection in Barbados. AIDS Res Hum Retroviruses. 2003;19(4):313-9.

81. Giuliani M, Montieri S, Palamara G, Latini A, Alteri C, Perno CF, et al. Non-B HIV type 1 subtypes among men who have sex with men in Rome, Italy. AIDS Res Hum Retroviruses. 2009;25(2):157-64.

82. González D, C M, Del A. Clinical, virological and phylogenetic characterization of a multiresistant HIV-1 strain outbreak in naive patients in southern Spain. Journal of Antimicrobial Chemotherapy. 2016;71(2):357-61.

83. Gonzalez-Domenech CM, Viciana I, Delaye L, Mayorga ML, Palacios R, de la Torre J, et al. Emergence as an outbreak of the HIV-1 CRF19_cpx variant in treatment-naive patients in southern Spain. PLoS One. 2018;13(1):e0190544.

84. Grgic I, Lepej SZ, Lunar MM, Poljak M, Vince A, Vrakela IB, et al. The prevalence of transmitted drug resistance in newly diagnosed HIV-infected individuals in Croatia: the role of transmission clusters of men who have sex with men carrying the T215S surveillance drug resistance mutation. AIDS Res Hum Retroviruses. 2013;29(2):329-36.

85. Grubb JR, Singhatiraj E, Mondy K, Powderly WG, Overton ET. Patterns of primary antiretroviral drug resistance in antiretroviral-naive HIV-1-infected individuals in a midwest university clinic. AIDS. 2006;20(16):2115-6.

86. Guanira J, Lama J, Montoya O, Segura P, Ramos E, Ganoza C, et al., editors. HIV drug resistance in the Andean Region: a look after the universal access to HAART in the Andean Region. ANTIVIRAL THERAPY; 2009: INT MEDICAL PRESS LTD 2-4 IDOL LANE, LONDON EC3R 5DD, ENGLAND.

87. Guimaraes ML, Marques BC, Bertoni N, Teixeira SL, Morgado MG, Bastos FI, et al. Assessing the HIV-1 Epidemic in Brazilian Drug Users: A Molecular Epidemiology Approach. PLoS One. 2015;10(11):e0141372.

88. Guo J, Yan Y, Zhang J, Ji J, Ge Z, Ge R, et al. Genetic characterization and antiretroviral resistance mutations among treatment-naive HIV-infected individuals in Jiaxing, China. Oncotarget. 2017;8(11):18271-9.

89. Hadas G. Transmission of HIV-1 drug-resistant mutations among treatment-naive infected patients in Tel-Aviv, Israel: Emergence of a cluster harbouring the major protease L90M mutation. Antiviral Therapy. 2010;15(3):A180.

90. Han ZG, Zhang YL, Wu H, Gao K, Zhao YT, Gu YZ, et al. [Prevalence of drug resistance in treatment-naive HIV infected men who have sex with men in Guangzhou, 2008-2015]. Zhonghua Liu Xing Bing Xue Za Zhi. 2018;39(7):977-82.

91. Hanna GJ, Balaguera HU, Freedberg KA, Werner BG, Steger Craven KA, Craven DE, et al. Drug-selected resistance mutations and non-B subtypes in antiretroviral-naive adults with established human immunodeficiency virus infection. J Infect Dis. 2003;188(7):986-91.

92. Hightow W, L B, C B, Ii G, T P, Enriquez B. Transmitted HIV-1 drug resistance among young men of color who have sex with men: A multicenter cohort analysis. Journal of Adolescent Health. 2011;48(1):94-9.

93. Hiransuthikul A, Wongkanya R, Sirivichayakul S, Trachunthong D, Sungsing T, Pankam T, et al. Short Communication: Discordance in Drug Resistance Mutations Between Blood Plasma and Semen or Rectal Secretions Among Newly Diagnosed HIV-1-Infected Thai Men Who Have Sex with Men. AIDS Res Hum Retroviruses. 2018;34(7):626-8.

94. Horban A, Stańczak J, Bąkowska E, Tobolewska E, Przybylska-Stengiel K, Stańczak G, et al. High prevalence of genotypic resistance to nucleoside reverse transcriptase inhibitors among therapy-naive individuals from the Warsaw cohort. Infection. 2002;30(6):356-9.

95. Hou LJ, Wang HW, Duan SP, Zhuo Y, Zhou YC, Wu HJ, et al. The prevalence and determinants of drug-resistance-associated mutations in the HIV-1-infected MSM population of Henan Province in China. Arch Virol. 2015;160(8):2051-61.

96. Hua J, Lin H, Ding Y, Qiu D, Wong F, He N. HIV drug resistance in newly diagnosed adults in a rural prefecture of eastern China. Epidemiol Infect. 2015;143(3):663-72.

97. Huaman MA, Aguilar J, Baxa D, Golembieski A, Brar I, Markowitz N. Late presentation and transmitted drug resistance mutations in new HIV-1 diagnoses in Detroit. Int J Infect Dis. 2011;15(11):e764-8.

98. Hurt CB, McCoy SI, Kuruc J, Nelson JA, Kerkau M, Fiscus S, et al. Transmitted antiretroviral drug resistance among acute and recent HIV infections in North Carolina from 1998 to 2007. Antivir Ther. 2009;14(5):673-8.

99. Iarikov DE, Irizarry-Acosta M, Martorell C, Hoffman RP, Skiest DJ. Low prevalence of primary HIV resistance in western Massachusetts. J Int Assoc Physicians AIDS Care (Chic). 2010;9(4):227-31.

100. Ibe S, Hotta N, Takeo U, Tawada Y, Mamiya N, Yamanaka K, et al. Prevalence of drug-resistant human immunodeficiency virus type 1 in therapy-naive patients and usefulness of genotype testing. Microbiol Immunol. 2003;47(7):499-505.

101. Iqbal HS, Solomon SS, Madhavan V, Solomon S, Balakrishnan P. Primary HIV-1 drug resistance and polymorphic patterns among injecting drug users (IDUs) in Chennai, Southern India. J Int Assoc Physicians AIDS Care (Chic). 2009;8(5):323-7.

102. Jayaraman GC, Archibald CP, Kim J, Rekart ML, Singh AE, Harmen S, et al. A population-based approach to determine the prevalence of transmitted drug-resistant HIV among recent versus established HIV infections: results from the Canadian HIV strain and drug resistance surveillance program. J Acquir Immune Defic Syndr. 2006;42(1):86-90.

103. Jeanette M, Michael J, Lynn E, An T, David A. Association Between Risk Behaviors and Antiretroviral Resistance in HIV-lnfected Patients Receiving Opioid Agonist Treatment. Journal of Addiction Medicine. 2013;7(2):102-7.

104. Jeong W, Jung IY, Choi H, Kim JH, Seong H, Ahn JY, et al. Integrase Strand Transfer Inhibitor Resistance Mutations in Antiretroviral Therapy-Naive and Treatment-Experienced HIV Patients in South Korea. AIDS Res Hum Retroviruses. 2019;35(2):213-6.

105. Jeulin H, Foissac M, Boyer L, Agrinier N, Perrier P, Kennel A, et al. Real-life rilpivirine resistance and potential emergence of an E138A-positive HIV strain in north-eastern France. J Antimicrob Chemother. 2014;69(11):3095-102.

106. Jiamsakul A, Sirivichayakul S, Ditangco R, Wong KH, Li PC, Praparattanapan J, et al. Transmitted drug resistance in recently infected HIV-positive Individuals from four urban locations across Asia (2007-2010) - TASER-S. AIDS Res Ther. 2015;12:3.

107. Jiamsakul A, Sungkanuparph S, Law M, Kantor R, Praparattanapan J, Li PC, et al. HIV multi-drug resistance at first-line antiretroviral failure and subsequent virological response in Asia. J Int AIDS Soc. 2014;17:19053.

108. Kanizsai S, Ghidan A, Ujhelyi E, Banhegyi D, Nagy K. Monitoring of drug resistance in therapy-naive HIV infected patients and detection of African HIV subtypes in Hungary. Acta Microbiol Immunol Hung. 2010;57(1):55-68.

109. Karade S, Patil AA, Ghate M, Kulkarni SS, Kurle SN, Risbud AR, et al. Short Communication: Limited HIV Pretreatment Drug Resistance Among Adults Attending Free Antiretroviral Therapy Clinic of Pune, India. AIDS Res Hum Retroviruses. 2016;32(4):377-80.

110. Karlsson A, Bjorkman P, Bratt G, Ekvall H, Gisslen M, Sonnerborg A, et al. Low prevalence of transmitted drug resistance in patients newly diagnosed with HIV-1 infection in Sweden 2003-2010. PLoS One. 2012;7(3):e33484.

111. Khan P, Apea V, DeMasi A, Kall M, Reeves I. Low prevalence of transmitted drug resistance (TDR) in an inner London genito-urinary medicine (GUM) clinic cohort with predominantly heterosexually transmitted, non-B-subtype infection: P151. Hiv Medicine. 2009;10.

112. Kotaki T, Khairunisa SQ, Witaningrum AM, Sukartiningrum SD, Diansyah MN, Rahayu RP, et al. HIV-1 transmitted drug resistance mutations among antiretroviral therapy-Naïve individuals in Surabaya, Indonesia. AIDS research and therapy. 2015;12(1):5.

113. Lai A, Violin M, Ebranati E, Franzetti M, Micheli V, Gismondo MR, et al. Transmission of resistant HIV type 1 variants and epidemiological chains in Italian newly diagnosed individuals. AIDS Res Hum Retroviruses. 2012;28(8):857-65.

114. Lan YC, Elbeik T, Dileanis J, Ng V, Chen YJ, Leu HS, et al. Molecular epidemiology of HIV-1 subtypes and drug resistant strains in Taiwan. J Med Virol. 2008;80(2):183-91.

115. Lapadula G, Izzo I, Gargiulo F, Paraninfo G, Castelnuovo F, Quiros-Roldan E, et al. Updated prevalence of genotypic resistance among HIV-1 positive patients naive to antiretroviral therapy: a single center analysis. J Med Virol. 2008;80(5):747-53.

116. Leszczyszyn P. Differences in the integrase and reverse transcriptase transmitted resistance patterns in Northern Poland. Infection, Genetics and Evolution. 2017;49:122-9.

117. Leszczyszyn P, Witak J, Bociaga J, Mozer L. Transmitted HIV drug resistance in antiretroviral-treatment-naive patients from Poland differs by transmission category and subtype. Journal of Antimicrobial Chemotherapy. 2015;70(1):233-42.

118. Li L, Han N, Lu J, Li T, Zhong X, Wu H, et al. Genetic characterization and transmitted drug resistance of the HIV type 1 epidemic in men who have sex with men in Beijing, China. AIDS research and human retroviruses. 2013;29(3):633-7.

119. Li L, Sun G, Liang S, Li J, Li T, Wang Z, et al. Different distribution of HIV-1 subtype and drug resistance were found among treatment naive individuals in Henan, Guangxi, and Yunnan province of China. PloS one. 2013;8(10).

120. Li L, Wei D, Hsu W-L, Li T, Gui T, Wood C, et al. CRF07_BC strain dominates the HIV-1 epidemic in injection drug users in Liangshan Prefecture of Sichuan, China. AIDS research and human retroviruses. 2015;31(5):479-87.

121. Li L, Wei D, Hsu WL, Li T, Gui T, Wood C, et al. CRF07_BC Strain Dominates the HIV-1 Epidemic in Injection Drug Users in Liangshan Prefecture of Sichuan, China. AIDS Res Hum Retroviruses. 2015;31(5):479-87.

122. Li X, Xue Y, Cheng H, Lin Y, Zhou L, Ning Z, et al. HIV-1 genetic diversity and its impact on baseline CD4+ T cells and viral loads among recently infected men who have sex with men in Shanghai, China. PloS one. 2015;10(6).

123. Liao L, Xing H, Shang H, Li J, Zhong P, Kang L, et al. The prevalence of transmitted antiretroviral drug resistance in treatment-naive HIV-infected individuals in China. Journal of acquired immune deficiency syndromes (1999). 2010;53 Suppl 1(Suppl 1):S10-S4.

124. Lindstrom A, Ohlis A, Huigen M, Nijhuis M, Berglund T, Bratt G, et al. HIV-1 transmission cluster with M41Lsingleton'mutation and decreased transmission of resistance in newly diagnosed Swedish homosexual men. Antiviral therapy. 2006;11(8):1031.

125. Lopes Maia T, Inácio B, M A, Lindenmeyer G, Gonçalves M. Trends in drug resistance mutations in antiretroviral-naïve intravenous drug users of Rio de Janeiro. Journal of Medical Virology. 2006;78(6):764-9.

126. Lu X, Kang X, Liu Y, Li Y, Chen S, Li J, et al. Surveillance of Transmitted Drug Resistance in HIV-1-Infected Youths Aged 16 to 25 Years, a Decade After Scale-up of Antiretroviral Therapy in Hebei, China. AIDS Res Hum Retroviruses. 2017;33(4):359-63.

127. Lunar MM, Židovec Lepej S, Abecasis AB, Tomažič J, Vidmar L, Karner P, et al. Prevalence of HIV type 1 transmitted drug resistance in Slovenia: 2005–2010. AIDS research and human retroviruses. 2013;29(2):343-9.

128. Maia Teixeira SL, Bastos FI, Hacker MA, Guimaraes ML, Morgado MG. Trends in drug resistance mutations in antiretroviral-naive intravenous drug users of Rio de Janeiro. J Med Virol. 2006;78(6):764-9.

129. Maljkovic I, Wilbe K, Solver E, Alaeus A, Leitner T. Limited transmission of drug-resistant HIV type 1 in 100 Swedish newly detected and drug-naive patients infected with subtypes A, B, C, D, G, U, and CRF01_AE. AIDS Res Hum Retroviruses. 2003;19(11):989-97.

130. Martínez G, Sarría L, Zubero Z, Santamaría J, Campelo M, Cisterna R. Deteccion de mutaciones asociadas a la resistencia a zidovudina en pacientes infectados por VIH. REVISTA ESPANOLA DE QUIMIOTERAPIA. 1997;10:54-60.

131. Masquelier B, Bhaskaran K, Pillay D, Gifford R, Balestre E, Jorgensen LB, et al. Prevalence of transmitted HIV-1 drug resistance and the role of resistance algorithms: data from seroconverters in the CASCADE collaboration from 1987 to 2003. J Acquir Immune Defic Syndr. 2005;40(5):505-11.

132. McFaul KM, Lim C, Jones R, Asboe D, Pozniak A, Sonecha S, et al. Transmitted antiretroviral resistance in a large HIV directorate 2011-2014: a response. AIDS. 2015;29(7):861-2.

133. Memarnejadian A, Menbari S, Mansouri SA, Sadeghi L, Vahabpour R, Aghasadeghi MR, et al. Transmitted drug resistance mutations in antiretroviral-naïve injection drug users with chronic HIV-1 infection in Iran. PloS one. 2015;10(5).

134. Mendoza Y, Castillo Mewa J, Martinez AA, Zaldivar Y, Sosa N, Arteaga G, et al. HIV-1 Antiretroviral Drug Resistance Mutations in Treatment Naive and Experienced Panamanian Subjects: Impact on National Use of EFV-Based Schemes. PLoS One. 2016;11(4):e0154317.

135. Metzner KJ, Rauch P, von Wyl V, Leemann C, Grube C, Kuster H, et al. Efficient suppression of minority drug-resistant HIV type 1 (HIV-1) variants present at primary HIV-1 infection by ritonavir-boosted protease inhibitor-containing antiretroviral therapy. J Infect Dis. 2010;201(7):1063-71.

136. Mezei M, Ay E, Koroknai A, Toth R, Balazs A, Bakos A, et al. Molecular epidemiological analysis of env and pol sequences in newly diagnosed HIV type 1-infected, untreated patients in Hungary. AIDS Res Hum Retroviruses. 2011;27(11):1243-7.

137. Morand-Joubert L, Frange P, Landman R, Kreplak G, Rouzioux C, Girard PM, et al., editors. Drug resistance and phylogenetic analysis of HIV-1 strains sampled from newly diagnosed untreated patients in Paris restricted area, France. International Workshop on HIV and Hepatitis Virus Drug Resistance and Curative Strategies; 2012 5-9 Jun, 2012; Sitge, Spain.

138. Moura ME, da Guarda Reis MN, Lima YA, Eulalio KD, Cardoso LP, Stefani MM. HIV-1 transmitted drug resistance and genetic diversity among patients from Piaui State, Northeast Brazil. J Med Virol. 2015;87(5):798-806.

139. Moura ME, Reis MN, Lima YA, Eulalio KD, Cardoso LP, Stefani MM. Low rate of transmitted drug resistance may indicate low access to antiretroviral treatment in Maranhao State, northeast Brazil. AIDS Res Hum Retroviruses. 2015;31(2):250-4.

140. Murillo W, Paz-Bailey G, Morales S, Monterroso E, Paredes M, Dobbs T, et al. Transmitted drug resistance and type of infection in newly diagnosed HIV-1 individuals in Honduras. Journal of Clinical Virology. 2010;49(4):239-44.

141. Ocfemia MCB, Saduvala N, Oster AM, Kim D, Kline R, Pearson M, et al. Transmitted HIV-1 drug resistance among men who have sex with men, 11 US jurisdictions, 2008–2011. Poster. 2014;213.

142. Oette M, Reuter S, Kaiser R, Lengauer T, Fatkenheuer G, Knechten H, et al. Epidemiology of transmitted drug resistance in chronically HIV-infected patients in Germany: the RESINA study 2001-2009. Intervirology. 2012;55(2):154-9.

143. Oliveira F, Lima V, J A. HIV-1 pol genetic diversity and antiretroviral resistance mutations in illicit drug users from Piaui, Northeast Brazil. Journal of the International AIDS Society. 2015;18:13-4.

144. Osman S, Lihana RW, Kibaya RM, Ishizaki A, Bi X, Okoth FA, et al. Diversity of HIV type 1 and drug resistance mutations among injecting drug users in Kenya. AIDS Res Hum Retroviruses. 2013;29(1):187-90.

145. P R, Dowdall S, Latendre P, R S. Follow-up investigation of a cluster of treatment-naive HIV-infected patients with multi-drug resistance in Sudbury, Ontario. Canadian Journal of Infectious Diseases and Medical Microbiology. 2013;24:38A.

146. Palma AC, Araujo F, Duque V, Borges F, Paixao M, Camacho R. Molecular epidemiology and prevalence of drug resistance-associated mutations in newly diagnosed HIV-1 patients in Portugal. Infection, Genetics and Evolution. 2007;7(3):391-8.

147. Pando M, Gómez-Carrillo M, Vignoles M, Rubio A, dos Ramos Farias MS, Vila M, et al. Incidence of HIV type 1 infection, antiretroviral drug resistance, and molecular characterization in newly diagnosed individuals in Argentina: A Global Fund Project. AIDS research and human retroviruses. 2011;27(1):17-23.

148. Pando MA, Eyzaguirre LM, Carrion G, Montano SM, Sanchez JL, Carr JK, et al. High genetic variability of HIV-1 in female sex workers from Argentina. Retrovirology. 2007;4:58.

149. Paraskevis D, Magiorkinis E, Katsoulidou A, Hatzitheodorou E, Antoniadou A, Papadopoulos A, et al. Prevalence of resistance-associated mutations in newly diagnosed HIV-1 patients in Greece. Virus Res. 2005;112(1-2):115-22.

150. Payne BA, Nsutebu EF, Hunter ER, Olarinde O, Collini P, Dunbar JA, et al. Low prevalence of transmitted antiretroviral drug resistance in a large UK HIV-1 cohort. J Antimicrob Chemother. 2008;62(3):464-8.

151. Pillay D, Bhaskaran K, Jurriaans S, Prins M, Masquelier B, Dabis F, et al. The impact of transmitted drug resistance on the natural history of HIV infection and response to first-line therapy. AIDS. 2006;20(1):21-8.

152. Pineda-Pena AC, Schrooten Y, Vinken L, Ferreira F, Li G, Trovao NS, et al. Trends and predictors of transmitted drug resistance (TDR) and clusters with TDR in a local Belgian HIV-1 epidemic. PLoS One. 2014;9(7):e101738.

153. Pontali E, Ventura A, Bruzzone B, Icardi G, Ferrari F. Unexpected high rate of wild-type HIV-1 genotype among inmates failing antiretroviral therapy. HIV Clin Trials. 2008;9(5):341-7.

154. Prellwitz IM, Alves BM, Ikeda ML, Kuhleis D, Picon PD, Jarczewski CA, et al. HIV behind bars: human immunodeficiency virus cluster analysis and drug resistance in a reference correctional unit from southern Brazil. PLoS One. 2013;8(7):e69033.

155. Prellwitz IM, Alves BM, Ikeda MLR, Kuhleis D, Picon PD, Jarczewski CA, et al. HIV behind bars: human immunodeficiency virus cluster analysis and drug resistance in a reference correctional unit from southern Brazil. PloS one. 2013;8(7).

156. Public Health Agency of Canada. HIV–1 strain and transmitted drug resistance in Canada: Surveillance report to December 31, 2008. Centre for Communicable Diseases and Infection Control, Public Health Agency of Canada. 2008.

157. Qi H, Zhao K, Xu F, Zhang X, Zhang Z, Yang L, et al. HIV-1 diversity, drug-resistant mutations, and viral evolution among high-risk individuals in phase II HIV vaccine trial sites in southern China. PLoS One. 2013;8(7):e68656.

158. Quang D, Nhan T, Yen N, Thuong V, Duc B, Thu Khanh H, et al. Pretreatment HIV-1 drug resistance to first-line drugs: results from a baseline assessment of a large cohort initiating ART in Vietnam, 2009-10. Journal of Antimicrobial Chemotherapy (JAC). 2015;70(3):941-7.

159. Resistance UKCGoMtToHD. Analysis of prevalence of HIV-1 drug resistance in primary infections in the United Kingdom. BMJ. 2001;322(7294):1087-8.

160. Reuter S, Oette M, Sichtig N, Kaiser R, Balduin M, Jensen B, et al. Changes in the HIV-1 mutational profile before first-line HAART in the RESINA cohort. J Med Virol. 2011;83(2):187-95.

161. Ristig MB, Arens MQ, Kennedy M, Powderly W, Tebas P. Increasing prevalence of resistance mutations in antiretroviral-naive individuals with established HIV-1 infection from 1996-2001 in St. Louis. HIV Clin Trials. 2002;3(2):155-60.

162. Riva C, Lai A, Caramma I, Corvasce S, Violin M, Deho L, et al. Transmitted HIV Type 1 drug resistance and Non-B subtypes prevalence among seroconverters and newly diagnosed patients from 1992 to 2005 in Italy. AIDS Res Hum Retroviruses. 2010;26(1):41-9.

163. Rossetti B, Di Giambenedetto S, Torti C, Postorino MC, Punzi G, Saladini F, et al. Evolution of transmitted HIV-1 drug resistance and viral subtypes circulation in Italy from 2006 to 2016. HIV Med. 2018;19(9):619-28.

164. Roudinskii NI, Sukhanova AL, Kazennova EV, Weber JN, Pokrovsky VV, Mikhailovich VM, et al. Diversity of human immunodeficiency virus type 1 subtype A and CRF03_AB protease in Eastern Europe: selection of the V77I variant and its rapid spread in injecting drug user populations. J Virol. 2004;78(20):11276-87.

165. Sagir A, Oette M, Kaiser R, Däumer M, Fätkenheuer G, Rockstroh JK, et al. Trends of prevalence of primary HIV drug resistance in Germany. Journal of antimicrobial chemotherapy. 2007;60(4):843-8.

166. Salama C, Ashraf A, editors. Comparison of the prevalence of HIV resistance mutations between US and foreign-born naive chronically infected patients New York City (NYC). ANTIVIRAL THERAPY; 2009: INT MEDICAL PRESS LTD 2-4 IDOL LANE, LONDON EC3R 5DD, ENGLAND.

167. Salama C, Karimjee J, editors. Prevalence of primary HIV resistance in an immigrant-rich HIV clinic in New York City. ANTIVIRAL THERAPY; 2011: INT MEDICAL PRESS LTD 2-4 IDOL LANE, LONDON EC3R 5DD, ENGLAND.

168. Sampathkumar R, Shadabi E, La D, Ho J, Liang B, Kimani J, et al. Naturally occurring protease inhibitor resistance mutations and their frequencies in HIV proviral sequences of drug-naïve sex workers in Nairobi, Kenya. Retrovirology. 2014;11(1):P133.

169. Sanabani SS, Pastena ER, da Costa AC, Martinez VP, Kleine-Neto W, de Oliveira AC, et al. Characterization of partial and near full-length genomes of HIV-1 strains sampled from recently infected individuals in Sao Paulo, Brazil. PLoS One. 2011;6(10):e25869.

170. Sapozhnikov J, Young JD, Patel M, Chiampas TD, Vaughn P, Badowski ME. Prevalence of HIV-1 transmitted drug resistance in the incarcerated population. HIV Med. 2017;18(10):756-63.

171. Shaw S, Pao D, Cane P, Badhan A, Koning F, Hobbs K, et al. HIV-1 Transmitted Drug Resistance (TDR) in Paired Plasma and Seminal Fluid: Persistence in Semen and Little Evidence of Differential Evolution. Hiv Medicine. 2012;13:8-9.

172. Shen Y, Su B, Wu J, Qin Y, Jin L, Miao L, et al. The prevalence of transmitted HIV drug resistance among MSM in Anhui province, China. AIDS Res Ther. 2014;11:19.

173. Shih Y-L, Hung Y-C, Chen W-F, Yang Y-T, Lai C-H, Lin H-H. The prevalence of genotype, risk groups, and drug-resistant to HAART in treatment naïve HIV-1 infected patients in southern Taiwan, 2004 to 2013. Journal of Microbiology, Immunology and Infection. 2015;48(2):S71.

174. Skoura L, Metallidis S, Buckton AJ, Mbisa JL, Pilalas D, Papadimitriou E, et al. Molecular and epidemiological characterization of HIV-1 infection networks involving transmitted drug resistance mutations in Northern Greece. J Antimicrob Chemother. 2011;66(12):2831-7.

175. Sprinz E, Netto EM, Patelli M, Lima JS, Furtado JJ, da Eira M, et al. Primary antiretroviral drug resistance among HIV type 1-infected individuals in Brazil. AIDS Res Hum Retroviruses. 2009;25(9):861-7.

176. Ssemwanga D, Ndembi N, Lyagoba F, Magambo B, Kapaata A, Bukenya J, et al. Transmitted antiretroviral drug resistance among drug-naive female sex workers with recent infection in Kampala, Uganda. Clin Infect Dis. 2012;54 Suppl 4:S339-42.

177. Stone DR, Corcoran C, Wurcel A, McGovern B, Quirk J, Brewer A, et al. Antiretroviral drug resistance mutations in antiretroviral-naive prisoners. Clin Infect Dis. 2002;35(7):883-6.

178. Sullivan PS, Buskin SE, Turner JH, Cheingsong R, Saekhou A, Kalish ML, et al. Low prevalence of antiretroviral resistance among persons recently infected with human immunodeficiency virus in two US cities. Int J STD AIDS. 2002;13(8):554-8.

179. Tamalet C, Tissot-Dupont H, Motte A, Tourres C, Dhiver C, Ravaux I, et al. Emergence of uncommon HIV-1 non-B subtypes and circulating recombinant forms and trends in transmission of antiretroviral drug resistance in patients with primary infection during the 2013-2015 period in Marseille, Southeastern France. J Med Virol. 2018;90(10):1559-67.

180. Taniguchi T, Nurutdinova D, Grubb JR, Onen NF, Shacham E, Donovan M, et al. Transmitted drug-resistant HIV type 1 remains prevalent and impacts virologic outcomes despite genotype-guided antiretroviral therapy. AIDS Res Hum Retroviruses. 2012;28(3):259-64.

181. Tanuma J, Quang VM, Hachiya A, Joya A, Watanabe K, Gatanaga H, et al. Low prevalence of transmitted drug resistance of HIV-1 during 2008–2012 antiretroviral therapy scaling up in Southern Vietnam. JAIDS Journal of Acquired Immune Deficiency Syndromes. 2014;66(4):358-64.

182. Todesco E, Charpentier C, Bertine M, Wirden M, Storto A, Desire N, et al. Disparities in HIV-1 transmitted drug resistance detected by ultradeep sequencing between men who have sex with men and heterosexual populations. HIV Med. 2017;18(9):696-700.

183. Torian LV, Forgione LA. Transmitted antiretroviral drug resistance in New York City, 2006-2010: the first five years of routine genotype surveillance. J Acquir Immune Defic Syndr. 2013;63(3):e119-22.

184. Tossonian HK, Raffa JD, Grebely J, Viljoen M, Mead A, Khara M, et al. Primary drug resistance in antiretroviral-naive injection drug users. Int J Infect Dis. 2009;13(5):577-83.

185. Tran VT, Ishizaki A, Nguyen CH, Hoang HTT, Pham HV, Bi X, et al. No increase of drug-resistant HIV type 1 prevalence among drug-naive individuals in Northern Vietnam. AIDS research and human retroviruses. 2012;28(10):1349-51.

186. Truong H, Kellogg T, Klausner J, Dilley J, Grant R, editors. HIV testing programmes as sentinel populations for HIV-1 drug resistance surveillance. ANTIVIRAL THERAPY; 2010: INT MEDICAL PRESS LTD 2-4 IDOL LANE, LONDON EC3R 5DD, ENGLAND.

187. Tsai HC, Chen IT, Wu KS, Tseng YT, Sy CL, Chen JK, et al. HIV-1 integrase strand-transfer inhibitor resistance in southern Taiwan. Oncotarget. 2018;9(38):24927-35.

188. Tupinambas U, Duani H, Martins AV, Aleixo AW, Greco DB. Transmitted human immunodeficiency virus-1 drug resistance in a cohort of men who have sex with men in Belo Horizonte, Brazil--1996-2012. Mem Inst Oswaldo Cruz. 2013;108(4):470-5.

189. Turner D, Amit S, Chalom S, Penn O, Pupko T, Katchman E, et al. Emergence of an HIV‐1 cluster harbouring the major protease L90M mutation among treatment‐naïve patients in T el A viv, I srael. HIV medicine. 2012;13(4):202-6.

190. Vercauteren J, Derdelinckx I, Sasse A, Bogaert M, Ceunen H, De Roo A, et al. Prevalence and epidemiology of HIV type 1 drug resistance among newly diagnosed therapy-naive patients in Belgium from 2003 to 2006. AIDS research and human retroviruses. 2008;24(3):355-62.

191. Violin M, Velleca R, Cozzi-Lepri A, Riva C, Grossi PA, Carnevale G, et al. Prevalence of HIV-1 primary drug resistance in seroconverters of the ICoNA cohort over the period 1996-2001. J Acquir Immune Defic Syndr. 2004;36(2):761-4.

192. Weinstock HS, Zaidi I, Heneine W, Bennett D, Garcia-Lerma GJ, Douglas Jr JM, et al. The epidemiology of antiretroviral drug resistance among drug-naive HIV-l-infected persons in 10 US cities. The Journal of infectious diseases. 2004;189(12):2174-80.

193. Weng YW, Tsai HC, Lee SS, Wu KS, Sy CL, Chen JK, et al. Prevalence and associated factors for HIV-1 transmitted drug resistance in voluntary clients for counseling and testing in Southern Taiwan. J Microbiol Immunol Infect. 2016;49(4):487-93.

194. Wensing AM, Vercauteren J, van de Vijver DA, Albert J, Asjo B, Balotta C, et al. Transmission of drug-resistant HIV-1 in Europe remains limited to single classes. Aids. 2008;22(5):625-35.

195. Wheeler WH, Ziebell RA, Zabina H, Pieniazek D, Prejean J, Bodnar UR, et al. Prevalence of transmitted drug resistance associated mutations and HIV-1 subtypes in new HIV-1 diagnoses, US–2006. Aids. 2010;24(8):1203-12.

196. Wong A, Kambo J, Harrigan R, Poon A, Joy JB, J B, editors. Large NNRTI-resistant transmission cluster in injection drug users from Saskatchewan. CROI; 2015 23-26 February, 2015; Seattle, Washington.

197. Xu Y, Peng X, Peng X, Ji S, Chen B, Wang L, et al. Characterization of HIV-1 subtypes and transmitted drug resistance among treatment-naive HIV-infected individuals in Zhejiang, China, 2014-2017. Arch Virol. 2018;163(8):2233-7.

198. Yan H, Ding Y, Wong FY, Ning Z, Zheng T, Nehl EJ, et al. Epidemiological and molecular characteristics of HIV infection among money boys and general men who have sex with men in Shanghai, China. Infection, Genetics and Evolution. 2015;31:135-41.

199. Yang C, Liu S, Zhang T, Hou Y, Liu X, Gao Y, et al. Transmitted antiretroviral drug resistance and thumb subdomain polymorphisms among newly HIV type 1 diagnosed patients infected with CRF01_AE and CRF07_BC virus in Guangdong Province, China. AIDS Res Hum Retroviruses. 2012;28(12):1723-8.

200. Yang J, Xing H, Niu J, Liao L, Ruan Y, He X, et al. The emergence of HIV-1 primary drug resistance genotypes among treatment-naive men who have sex with men in high-prevalence areas in China. Archives of virology. 2013;158(4):839-44.

201. Yang WL, Kouyos R, Scherrer AU, Boni J, Shah C, Yerly S, et al. Assessing the Paradox Between Transmitted and Acquired HIV Type 1 Drug Resistance Mutations in the Swiss HIV Cohort Study From 1998 to 2012. J Infect Dis. 2015;212(1):28-38.

202. Ye J, Guo L, Lu H, Xin R, Zeng Y. Genotypic resistance mutations to antiretroviral drugs in newly confirmed human immunodeficiency virus infectors in Beijing. Zhonghua yi xue za zhi. 2011;91(21):1453-6.

203. Ye JR, Lu HY, Wang WS, Guo L, Xin RL, Yu SQ, et al. The prevalence of drug resistance mutations among treatment-naive HIV-infected individuals in Beijing, China. AIDS Res Hum Retroviruses. 2012;28(4):418-23.

204. Yebra G, de Mulder M, Pérez-Elías MJ, Pérez-Molina JA, Galán JC, Llenas-Garcia J, et al. Increase of transmitted drug resistance among HIV-infected sub-Saharan Africans residing in Spain in contrast to the native population. PLoS One. 2011;6(10).

205. Yebra G, Delgado R, Pulido F, Rubio R, Galán JC, Moreno S, et al. Different trends of transmitted HIV-1 drug resistance in Madrid, Spain, among risk groups in the last decade. Archives of virology. 2014;159(5):1079-87.

206. Yebra G, Holguin A, Pillay D, Hue S. Phylogenetic and demographic characterization of HIV-1 transmission in Madrid, Spain. Infect Genet Evol. 2013;14(1):232-9.

207. Youmans E, Tripathi A, Albrecht H, Gibson JJ, Duffus WA. Transmitted antiretroviral drug resistance in individuals with newly diagnosed HIV infection: South Carolina 2005-2009. South Med J. 2011;104(2):95-101.

208. Yu G, Li Y, Li J, Diao L, Yan X, Lin P, et al. Genetic diversity and drug resistance of HIV type 1 circulating recombinant Form_BC among drug users in Guangdong Province. AIDS research and human retroviruses. 2009;25(9):869-75.

209. Zarandia M, Tsertsvadze T, Carr JK, Nadai Y, Sanchez JL, Nelson K. HIV-1 genetic diversity and genotypic drug susceptibility in the Republic of Georgia. AIDS Research & Human Retroviruses. 2006;22(5):470-6.

210. Zhang J, Guo Z, Pan X, Zhang W, Yang J, Ding X, et al. Highlighting the crucial role of Hangzhou in HIV-1 transmission among men who have sex with men in Zhejiang, China. Sci Rep. 2017;7(1):13892.

211. Zhang J, Guo Z, Yang J, Pan X, Jiang J, Ding X, et al. Genetic diversity of HIV-1 and transmitted drug resistance among newly diagnosed individuals with HIV infection in Hangzhou, China. J Med Virol. 2015;87(10):1668-76.

212. Zhang J, Yang J, Pan X, Guo Z, Ding X, Xu Y, et al. [HIV-1 subtype diversity and transmission clusters among men having sex with men who recently got HIV-l infection, in Zhejiang province]. Zhonghua Liu Xing Bing Xue Za Zhi. 2015;36(1):61-6.

213. Zhang X, Li S, Li X, Li X, Xu J, Li D, et al. Characterization of HIV-1 subtypes and viral antiretroviral drug resistance in men who have sex with men in Beijing, China. AIDS. 2007;21 Suppl 8:S59-65.

214. Zhao B, Han X, Dai D, Liu J, Ding H, Xu J, et al. New trends of primary drug resistance among HIV type 1-infected men who have sex with men in Liaoning Province, China. AIDS Res Hum Retroviruses. 2011;27(10):1047-53.

215. Zhao B, Han X, Xu J, Hu Q, Chu Z, Zhang J, et al. Increase of RT-related transmitted drug resistance in non-CRF01_AE among HIV type 1-infected men who have sex with men in the 7 cities of China. J Acquir Immune Defic Syndr. 2015;68(3):250-5.

216. Zhao GL, Yu W, Cai YM, Wang F, Hong FC, Jiang WN, et al. [Primary drug resistance of HIV-1 infected men who have sex with men in Shenzhen, China]. Zhonghua Yi Xue Za Zhi. 2012;92(17):1165-9.

217. Zhong P, Kang L, Pan Q, Konings F, Burda S, Ma L, et al. Identification and distribution of HIV type 1 genetic diversity and protease inhibitor resistance-associated mutations in Shanghai, P. R. China. J Acquir Immune Defic Syndr. 2003;34(1):91-101.

218. Zuckerman NS, Mor Z, Bucris E, Wax M, Mendelson E, Mor O. Sexual intermingling of Arab and Jewish MSM in Israel: results of a molecular epidemiology study. AIDS. 2019;33(2):339-44.
